# Molecular phylogeny and taxonomic revision of the sportive lemurs (*Lepilemur*, Primates)

**DOI:** 10.1186/1471-2148-6-17

**Published:** 2006-02-23

**Authors:** Nicole Andriaholinirina, Jean-Luc Fausser, Christian Roos, Dietmar Zinner, Urs Thalmann, Clément Rabarivola, Iary Ravoarimanana, Jörg U Ganzhorn, Bernhard Meier, Roland Hilgartner, Lutz Walter, Alphonse Zaramody, Christoph Langer, Thomas Hahn, Elke Zimmermann, Ute Radespiel, Mathias Craul, Jürgen Tomiuk, Ian Tattersall, Yves Rumpler

**Affiliations:** 1 Institut d'Embryologie, Université Louis Pasteur, Faculté de Médecine-EA3428, 11 rue Humann, 67085 Strasbourg, France; 2 Faculté des Sciences Dépt. d'Anthropologie Biologique, Antananarivo, Madagascar; 3 Primate Genetics, Deutsches Primatenzentrum, Kellnerweg 4, 37077 Göttingen, Germany; 4 Gene Bank of Primates, Deutsches Primatenzentrum, Kellnerweg 4, 37077 Göttingen, Germany; 5 Cognitive Ethology, Deutsches Primatenzentrum, Kellnerweg 4, 37077 Göttingen, Germany; 6 Anthropological Institute, University of Zürich, Winterthurerstr. 190, 8057 Zürich, Switzerland; 7 Faculté des Sciences de Mahajanga, Mahajanga, Madagascar; 8 Kölnerstr. 88, 57368 Grevenbrück, Germany; 9 Abt. Tierökologie und Naturschutz, Biozentrum Grindel, Universität Hamburg, Hamburg, Germany; 10 Behavioral Ecology and Sociobiology, Deutsches Primatenzentrum, Kellnerweg 4, 37077 Göttingen, Germany; 11 Université de Mahajanga, Faculté des Sciences, Dépt. de Biologie Animale, B.P. 652, Mahajanga 401, Madagascar; 12 von Freybergstr. 45, 87629 Füssen, Germany; 13 Dept. of Cell Physiology, Max-Planck-Institute for Medical Research, Jahnstr. 29, 69120 Heidelberg, Germany; 14 Institute of Zoology, University of Veterinary Medicine, Hannover, Bünteweg 17, 30559 Hannover, Germany; 15 Institute of Human Genetics, University of Tübingen, Wilhelmstr. 27, 72074 Tübingen, Germany; 16 Division of Anthropology, American Museum of Natural History New York, New York 10024, USA

## Abstract

**Background:**

The number of species within the Malagasy genus *Lepilemur *and their phylogenetic relationships is disputed and controversial. In order to establish their evolutionary relationships, a comparative cytogenetic and molecular study was performed. We sequenced the complete mitochondrial cytochrome b gene (1140 bp) from 68 individuals representing all eight sportive lemur species and most major populations, and compared the results with those obtained from cytogenetic studies derived from 99 specimens.

**Results:**

Interspecific genetic variation, diagnostic characters and significantly supported phylogenetic relationships were obtained from the mitochondrial sequence data and are in agreement with cytogenetic information. The results confirm the distinctiveness of *Lepilemur ankaranensis*, *L. dorsalis*, *L. edwardsi*, *L. leucopus*, *L. microdon*, *L. mustelinus*, *L. ruficaudatus *and *L. septentrionalis *on species level. Additionally, within *L. ruficaudatus *large genetic differences were observed among different geographic populations. *L. dorsalis *from Sahamalaza Peninsula and from the Ambanja/Nosy Be region are paraphyletic, with the latter forming a sister group to *L. ankaranensis*.

**Conclusion:**

Our results support the classification of the eight major sportive lemur taxa as independent species. Moreover, our data indicate further cryptic speciation events within *L. ruficaudatus *and *L. dorsalis*. Based on molecular data we propose to recognize the sportive lemur populations from north of the Tsiribihina River, south of the Betsiboka River, and from the Sahamalaza Peninsula, as distinct species.

## Background

**Figure 1:**
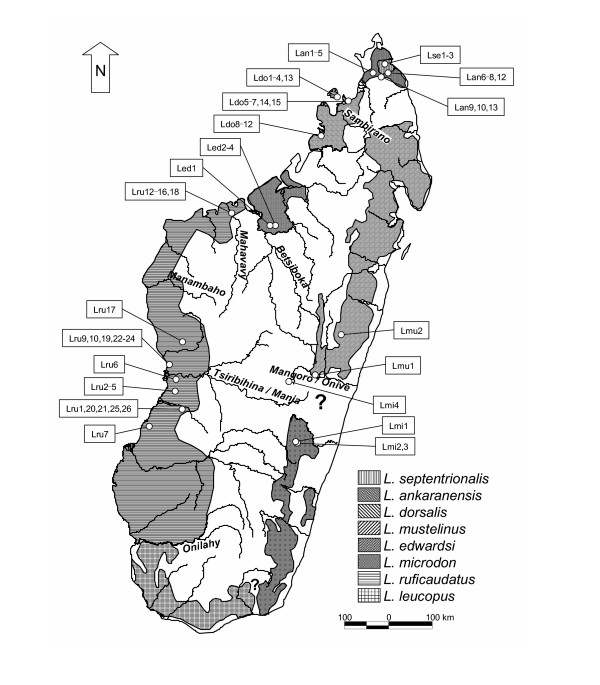
Distribution of *Lepilemur *species (based on [25]). Circles indicate origin of analysed individuals (abbreviations are listed in additional file 1). Labelled rivers represent possible biogeographical barriers. Question marks indicate areas with ambiguous or missing information on *Lepilemur *distribution.

Sportive lemurs, genus *Lepilemur*, are small nocturnal primates endemic to the island of Madagascar. They are amongst the most widely distributed lemurs, occurring in almost all natural evergreen or deciduous forest formations on the island (Fig. 1). Because pelage colouration and other external characteristics are inconspicuous in sportive lemurs, the early classifications [1,2] based on these features were disputed until a comprehensive cytogenetic approach allowed the recognition of six species [3-6]: *L. dorsalis*, *L. edwardsi*, *L. leucopus*, *L. mustelinus L. ruficaudatus *and *L. septentrionalis*. For a seventh, *L. microdon*, the karyotype remained unknown. Recently, *L. septentrionalis *has been split into two separate species, *L. septentrionalis *and *L. ankaranensis *[7,8] and the karyotype of *L. microdon *has been established [9], so that there are now eight cytogenetically recognized species.

Molecular studies, especially the sequencing of mitochondrial genes, have expanded enormously during the last decade and have helped to characterise biodiversity and biogeographic patterns of Malagasy lemurs [10-19]. Such studies have been particularly fruitful among the family Cheirogaleidae, in which several new mouse lemur species were described and others elevated from synonymy [11,18,20], despite the fact that no chromosomal differences were detected within the subfamily Cheirogaleinae [21-23].

The application of mitochondrial sequence data to the reconstruction of phylogenetic relationships within the genus *Lepilemur *is still rare. However, pioneering studies using this method have helped to solve taxonomic issues. Some of the results have confirmed previous conclusions such as the species status of *L. septentrionalis *and *L. ankaranensis *[24], while other studies revealed unexpected results such as the paraphyly of *L. edwardsi *from south and north of the Betsiboka River [12,17], suggesting the existence of two different species [6,12,17]. Within *L. ruficaudatus*, Roos [25] also detected a paraphyly, with specimens from Andramasay, north of the Tsiribihina River, being more closely related to *L. edwardsi *from south of the Betsiboka River than they are to *L. ruficaudatus *from Kirindy, south of the Tsiribihina. The classification of specimens from south of the Betsiboka as *L. edwardsi *was, however, based on erroneous information on the extension of the species' range as far south as the Tsiribihina [3,5], despite the report of the existence of *L. ruficaudatus *north of the Tsiribihina in the Antsalova region [4]. Later, sportive lemurs south of the Betsiboka were cytogenetically classified as *L. ruficaudatus *[26].

In order to obtain a more complete picture of the sportive lemur's evolution, diversity and biogeography, we conducted a comparative molecular study by combining mitochondrial sequence data with cytogenetic information from all currently recognized species. We sequenced the complete mitochondrial cytochrome b gene from 68 individuals from 21 geographic locations, and compared the obtained results with conclusions from cytogenetic data derived from 99 individuals.

## Results

### Cytogenetics

Karyograms were established for 99 individuals during studies performed between 1975 and 2005, with one to 28 specimens from each study population (see additional files 1 and 2). As shown in additional file 2, the diploid chromosome number within the genus ranges from 20 to 38, and one to 19 chromosomal rearrangements were detected between species. Among the eight recognized species, karyotypes are species specific. Among the different populations of the traditional *L. ruficaudatus *and *L. dorsalis *no differences in diploid chromosome number and in the R-banding pattern were detected (see additional file 2, Fig. 2, 3, 4). Only one *L. ruficaudatus *from Anjahamena was analyzed by Giemsa staining and this specimen showed a karyotype identical to that of other *L. ruficaudatus *(Fig. 4). Further details about diploid chromosome numbers for all species and populations, and rearrangements among them, are given in additional file 2.

**Figure 2:**
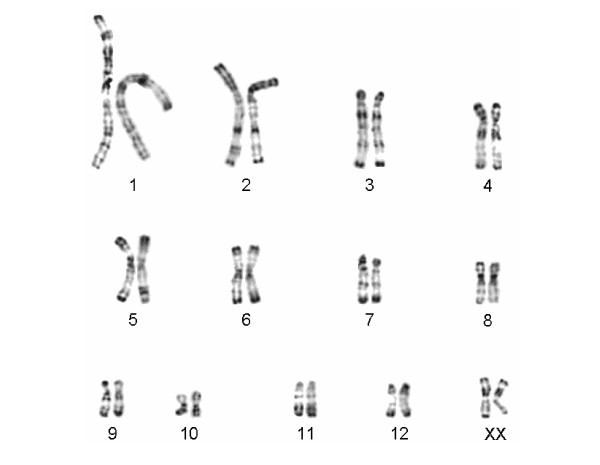
Comparison of the half karyotypes of *Lepilemur dorsalis *from Sahamalaza Peninsula (left) and Ambanja (right). No differences in the R-banding pattern were detected.

**Figure 3:**
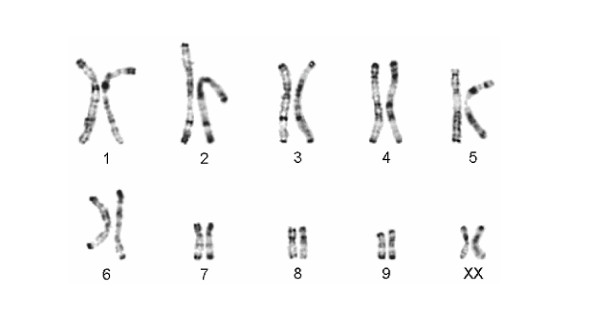
Comparison of the half karyotypes of *Lepilemur ruficaudatus *from Andramasay (left) and Kirindy/CFPF (right). No differences in the R-banding pattern were detected.

**Figure 4:**
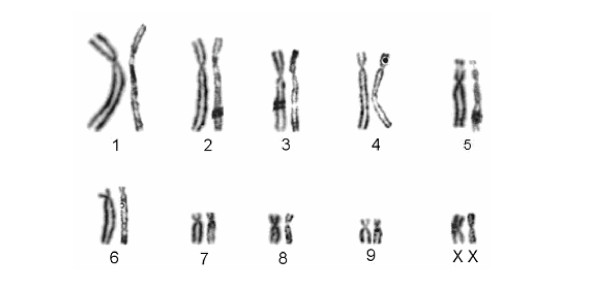
Comparison of the half karyotypes of *Lepilemur ruficaudatus *from Kirindy/CFPF (left) and Anjahamena (right). No differences in the Giemsa-stained karyotypes were detected.

### Molecular genetics

Complete mitochondrial cytochrome b gene sequences (1140 bp) were generated from 68 individuals representing all currently recognized species. Among the 68 sequences we detected 39 haplotypes. Uncorrected pairwise differences within the genus range from 0.00–16.82%, with overlapping intra- and inter-specific differences (intra-specific: 0.00–7.63%, inter-specific: 2.90–16.82%). Although most inter-specific differences are in the range of 7.37–16.82%, there are also two exceptions which show that differences between species can be much lower (*L. ankaranensis *– *L. dorsalis *from Sahamalaza Peninsula: 4.56–5.35%; *L. ankaranensis *– *L. dorsalis *from Ambanja/Nosy Be: 2.90–3.60%). Intra-specific differences range from 0.00–7.63%. Interestingly, the observed differences of 5.88–7.63% between the three major populations of *L. ruficaudatus*, which are separated by wide rivers or large distances, and those found between *L. dorsalis *from Sahamalaza Peninsula and from Ambanja/Nosy Be (5.18–5.88%) exceed those detected between *L. ankaranensis *and the two *L. dorsalis *populations, indicating speciation events in *L. ruficaudatus *and *L. dorsalis*. Further details about pairwise differences within and between species are reported in additional file 3.

Based on the 1140 bp of the mitochondrial cytochrome b gene, a population aggregation analysis was performed with 373 positions serving as diagnostic characters (see additional file 4). With 163–183 diagnostic characters, *L. mustelinus *is clearly separated from the other species. The remaining species differ in 32–146 diagnostic characters, with the lowest detected between *L. ankaranensis *and *L. dorsalis *from the Ambanja/Nosy Be population. The three *L. ruficaudatus *populations are separated by 64–78 diagnostic characters, comparable to those distinguishing the two *L. dorsalis *populations (57 characters), or to those detected between the two widely recognized species *L. septentrionalis *and *L. ankaranensis *(82 characters).

**Figure 5:**
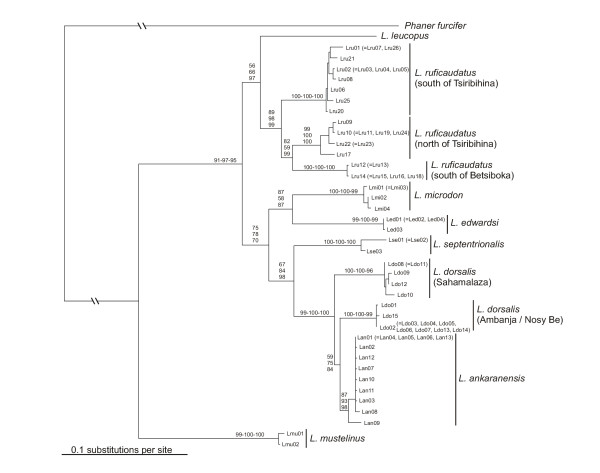
Phylogenetic relationships as obtained from complete mitochondrial cytochrome b gene sequences (39 haplotypes), with branch lengths drawn according to those estimated by the NJ algorithm and by applying the TVM + I + Γ model of sequence evolution. Abbreviations refer to those listed in additional file 1 and numbers on nodes indicate support values for internal branches (first: NJ, second: MP, third: ML) based on 1,000 bootstrap replicates (NJ, MP) or 10,000 quartet puzzling steps (ML).

Phylogenetic trees reconstructed on the basis of different algorithms and models of sequence evolution produced identical tree topologies, with mainly significantly supported branching patterns (Fig. 5). Based on inferred phylogenetic relationships, *L. mustelinus *was the first species to split off. The remaining species diverged into two subgroups, of which one contained *L. ankaranensis, L. dorsalis, L. edwardsi, L. microdon *and *L. septentrionalis*, and the other *L. leucopus *and *L. ruficaudatus*. Within *L. ruficaudatus *three very distinct clades were observed, corresponding to different geographic locations. In the other subgroup a major split occurred between *L. microdon/L. edwardsi *and the remaining species, which later separated into *L. septentrionalis *and a group consisting of *L. dorsalis *and *L. ankaranensis*. *L. dorsalis *is further divided into two subclades, with one containing individuals from Ambanja and Nosy Be, and the other those from Sahamalaza. Moreover, *L. dorsalis *emerges as paraphyletic, with specimens from Nosy Be/Ambanja forming a sister clade to *L. ankaranensis*.

## Discussion

As in many nocturnal mammals, pelage colouration among sportive lemurs is not a suitable characteristic for distinguishing among taxa, since it is not well defined and consistent within taxa. In contrast, cytogenetics or molecular methods such as sequencing of marker genes offer powerful tools that lead to important insights into the diversity and phylogeny of several Malagasy lemur genera [17,19,28,29]. In order to determine the diversity and phylogenetic relationships among sportive lemur taxa we conducted a comparative molecular study by combining mitochondrial sequence information and cytogenetic data from all currently recognized *Lepilemur *species.

Based on cytogenetic data, the genus *Lepilemur *can be divided into the eight traditionally recognized species, *L. ankaranensis*, *L. dorsalis*, *L. edwardsi*, *L. leucopus*, *L. microdon*, *L. mustelinus*, *L. ruficaudatus *and *L. septentrionalis*. These differ by several chromosomal rearrangements (3 to 19), with the exception of *L. septentrionalis *and *L. ankaranensis *that only one rearrangement separates [7-9] (see additional file 2). Nevertheless, the phylogenetic positions as well the genetic distances separating these two latter species suggest a separation on species level [24]. Among the three *L. ruficaudatus *populations and the two *L. dorsalis *populations, no chromosomal differences were detected.

The mitochondrial sequence data also confirm the specific status of the eight species mentioned above. The genetic differences detected among them are in the range of those observed among species of other lemur genera such as *Mirza *[11], *Microcebus *[11,14,18], *Hapalemur *[10,16] and *Propithecus *[13,30]. Interestingly, the distance of 14.47–16.82% observed between *L. mustelinus *and the remaining *Lepilemur *species is the largest detected among lemur species to date, and exceeds those observed among mouse lemur species [11,18].

Besides the high genetic differences observed among the eight *Lepilemur *species, further large differences were detected within *L. ruficaudatus *and *L. dorsalis*. The molecular data strongly indicate that the three *L. ruficaudatus *populations that are distributed in distinct subclades and separated by high genetic differences of 5.88–7.63% represent different taxa, confirming previous suggestions of a separate taxon status for specimens from Kirindy, Andramasay and Anjahamena [6,12,17,25,31]. Although no chromosomal differences were detected among the three *L. ruficaudatus *populations, the genetic differences observed among them are higher than those detected among some other *Lepilemur *species (e.g. *L. dorsalis *– *L. ankaranensis*: 2.90–3.60%), as well as from comparable data from other lemur genera, e.g. *Microcebus *[11,18]. Thus a separation of the three populations at the species level is proposed, which is additionally supported by the fact that these populations occupy ranges between major biogeographical barriers, such as Madagascar's large rivers (Fig. 1). These rivers are barriers also for a number of other species (Tsiribihina: *Geogale aurita*, *Echinops telfairi*, *Hypogeomys antimena*, *Mungotictis decemlineata*, *Propithecus verreauxi verreauxi*, *Microcebus berthae*; Mahavavy: *Eulemur fulvus rufus*, *Galida elegans occidentalis*; Betsiboka: *Oryzorictes talpoides*, *Avahi occidentalis*; Sambirano: *Phaner furcifer parienti *and possibly *Avahi unicolor*).

The *L. dorsalis *populations of Ambanja and Nosy Be are closely related and form one clade, indicating both their common origin and a recent isolation of the island of Nosy Be. The Nosy Be/Ambanja *L. dorsalis *and *L. ankaranensis *together form a sister group to *L. dorsalis *from Sahamalaza, indicating a paraphyly of *L. dorsalis*. These phylogenetic and biogeographic patterns are also confirmed by RAPD analysis [32], and are similar to patterns obtained for the genus *Propithecus *(Indriidae) in that *P. v. coquereli *appears to cluster with *P. tattersalli *instead with other subspecies of *P. verreauxi *[13,17,25,30]. Although ten chromosomal rearrangements were detected between *L. ankaranensis *and Nosy Be/Ambaja *L. dorsalis *(see additional file 2), genetic differences are, at 2.90–3.60%, the lowest detected among all *Lepilemur *species (see additional file 3). *L. dorsalis *from Sahamalaza differs from the Nosy Be/Ambanja *L. dorsalis *and *L. ankaranensis *at 4.56–5.88%, which is comparable with differences among other *Lepilemur *species. Taking together the paraphyly of *L. dorsalis *and the large genetic differences among analysed populations, we propose to divide the traditional *L. dorsalis *into two distinct species.

Furthermore, the distribution of diagnostic characters derived from a PAA also suggests a split of the *L. ruficaudatus *population into three different taxa and the *L. dorsalis *population into two taxa. This pattern is congruent with the results of the pairwise distance comparison and the phylogenetic tree reconstruction.

Since the type locality of *L. ruficaudatus *and its synonym *L. pallidicauda *is simply "Morondava" and no other synonyms are available, the populations north of the Tsiribihina River and between the rivers Betsiboka and Mahavavy need to be named. Questionable, however, is the classification of another new species announced by Edward Louis from Mitsinjo [33], a village located on the south side of the Mahavavy du Sud River. Whether this species is the same as the one we describe from the region between the Betsiboka and Mahavavy rivers needs to be evaluated by further studies. The locality of the *L. dorsalis *type specimen is very imprecisely labelled as "Madagascar". The only possible synonym for *L. dorsalis *is "*Lepidolemur" grandidieri*, with the similarly imprecise origin "North-western Madagascar". Most museum specimens are from the Ambanja region (see also [11]), and hence we presume that this is also the case for the *L. dorsalis *and *L. grandidieri *holotypes. Therefore, the population from the Sahamalaza Peninsula needs to be named. Below, we describe the sportive lemurs from the regions between the Betsiboka and Mahavavy rivers, north of the Tsiribihina River, and the Sahamalaza Peninsula, as three new species:

### *Lepilemur aeeclis *sp. nov. (Fig. 6)

**Figure 6:**
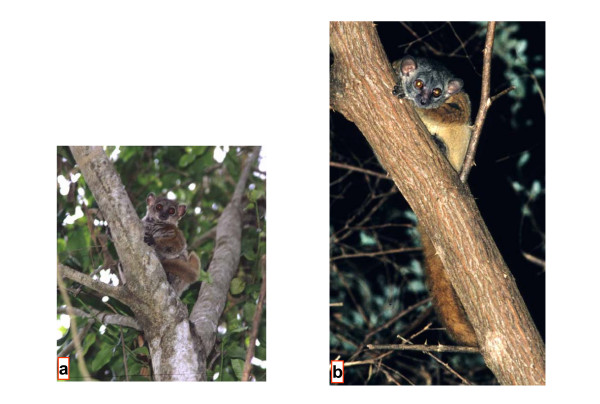
*L. aeeclis *from Anjahamena during the day (a) and night (b) (Photographs by U. Thalmann).

#### Type Series

Skull (UM 2003-Lem-100, see additional file 5) stored at the University of Mahajanga. The individual was found dead at the type locality. Hair and DNA samples from an additional five different individuals are stored at the University Louis Pasteur Strasbourg, France and the Gene Bank of Primates, German Primate Centre, Germany (GBP 1028-1032). Measurements for individuals are given in additional file 6.

#### Type locality

Antafia (approx. 16°03.057'S, 45°54.522'E), north-east side of the Mahavavy du Sud River, Fokotany Ambatomahavavy, Firaisana Antongomena-Bevary, Fivondronona Mitsinjo, Province Mahajanga, Madagascar.

#### Description

Pelage coloration is considerably variable in the expression of the colours, possibly as a function of the age of individuals. Depending on light conditions (daylight or flashlight at night) the impression of colours may change subjectively. However, some constant characters are present, though variably expressed. The face is essentially grey and the ears are protruding and rounded. Sometimes there is the impression of a "facemask", in that there may be a darker diffuse, patch of hair in the middle of the forehead. Above the eyes darker coloured but diffuse stripes may run upwards to join in the middle of the head. These confluent stripes continue as one darker and distinct stripe along the back. The stripe is especially well expressed until it reaches the middle of the back, and then continues less prominently to the tail. On the back, the animals are essentially grey and reddish grey. The middle part of the back, especially, may show considerable reddish colouration that extends onto both shoulders and the upper and lower arms. The thigh and lower limb in general are less reddish than the upper part of the body. The ventral pelage is light to darker grey. The tail is variably coloured between grey with some red influence to deep rusty red with negligible grey influence.

#### Diagnosis

Differs, with the exception of *L. randrianasoli *and *L. ruficaudatus*, from all other sportive lemurs in karyotype (2N = 20, Fig. 4, see additional file 2). In the complete mitochondrial cytochrome b gene, *L. aeeclis *differs from its closest relative, *L. randrianasoli *by 5.88–6.75% and in 64 diagnostic characters. It is slightly larger than *L. randrianasoli *but is similar in size to *L. ruficaudatus *(see additional file 6). Head measurements (length and width) are more similar to *L. ruficaudatus *than to *L. randrianasoli*. However, hind foot length is more similar to *L. randrianasoli *than to *L. ruficaudatus*, which has the shortest hind foot.

#### Etymology

*L. aeeclis *is named in honour of the Association Européenne pour l'Etude et la Conservation des Lémuriens (A.E.E.C.L.), which has supported our fieldwork for 12 years.

#### Distribution

The taxon occurs between the Betsiboka and Mahavavy du Sud rivers. The southern extension of the taxon across the Mahavavy du Sud River is unknown, and needs further research.

### *Lepilemur randrianasoli *sp. nov. (Fig. 7)

**Figure 7:**
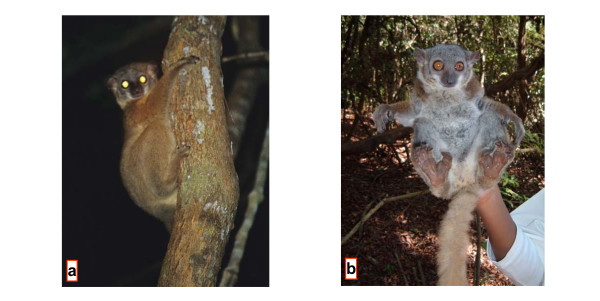
*L. randrianasoli *from the southern bank of Manambolo River, approx. 35 km NE of type location (a) and from Ambalarano (b) (Photographs by U. Thalmann (a) and N. Andriaholinirina (b)).

#### Holotype

Tissue and DNA from one individual stored at the Gene Bank of Primates, German Primate Centre, Germany (GBP 941).

#### Type locality

Andramasay (approx. 44°29'E, 19°28'S), Province Toliary, Madagascar.

#### Description

Measurements of five males and four females from the type locality Andramasay are listed in additional file 6.

#### Diagnosis

Differs, with the exception of *L. aeeclis *and *L. ruficaudatus*, from all other sportive lemurs in karyotype (2N = 20, Fig. 3, see additional file 2). In the complete mitochondrial cytochrome b gene, *L. randrianasoli *differs from its closest relative, *L. aeeclis*, by 5.88–6.75% and in 64 diagnostic characters. The species is slightly smaller than *L. aeeclis *and *L. ruficaudatus *(see additional file 5). It differs from these species by having a narrower but slightly longer head. These differences in head size are most pronounced in males. Hind feet are of similar length to *L. aeeclis*, but longer than in *L. ruficaudatus*. Tail length is similar in all three species.

#### Etymology

*L. randrianasoli *is named in honour of our late colleague, Georges Randrianasolo, who worked from 1970 to 1977 to sample the sportive lemurs necessary for the first taxonomic revision based on cytogenetic studies, and who walked for two weeks to obtain data and samples from the *L. ruficaudatus *from Antsalova.

#### Distribution

Currently, *L. randrianasoli *is restricted to the type locality and Bemaraha. The Tsiribihina River is most likely the southern limit of the species' range. Further fieldwork is necessary to determine the northern limit. A possible northern barrier could be one of the major rivers Manambaho or the Mahavavy du Sud.

### *Lepilemur sahamalazensis *sp. nov. (Fig. 8)

**Figure 8:**
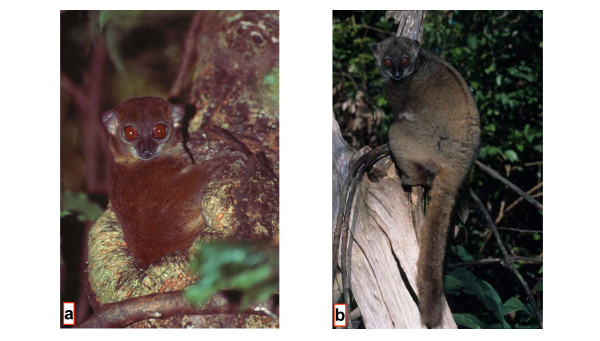
*L. sahamalazensis *at its type locality Sahamalaza Peninsula (Photographs by T. Hahn (a) and R. Hilgartner (b)).

#### Type Series

Tissue and DNA samples from 4 individuals stored at the University Louis Pasteur Strasbourg, France (Ldo158s, Ldo153s, Ldo40, Lepi205Ak99).

#### Type locality

Sahamalaza Peninsula (approx. 47°58'E, 14°16'S), Province Mahajanga, Madagascar. Hypodigm: 6 animals from Ankarafa (47°45'E, 14°22'S), 25 km southeast of the type locality, for which morphometric measurements have been taken.

#### Description

Pelage coloration is variable, possibly also depending on age of individuals. Depending on light conditions (daylight or flashlight at night) the impression of colours may change subjectively. However, some constant characters are present, though variably expressed. The face is essentially grey. The forehead and the hairline around the ears are red-brown with sometimes darker diffuse patches. A dark diffuse line runs from the middle of the upper skull down the spine, ending in the middle or at the lower part of the back, but is never present on the tail. The dorsal pelage, including shoulders and the upper and lower arms, is predominantly red-brown, whereas the thigh and lower limbs in general are less reddish than the upper part of the body. The ventral pelage is generally grey to creamy. The coloration of the tail is red-brown to deep brown. Measurements of 2 males and of 4 females from Ankarafa are listed in additional file 7.

#### Diagnosis

Differs, with the exception of *L. dorsalis *and *L. leucopus*, from all other sportive lemurs in diploid chromosome number (2N = 26 Fig. 2, see additional file 2). The karyotypes of *L. leucopus *and *L. sahamalazensis *differ in six chromosomal rearrangements, whereas none exists between the latter and *L. dorsalis*. In the complete mitochondrial cytochrome b gene, *L. sahamalazensis *differs from its closest relatives, *L. dorsalis *and *L. ankaranensis*, in 5.18–5.88% and 4.56–5.35% and by 57 and 50 diagnostic characters, respectively. The few morphometric data which are available at the moment indicate that *L. sahamalazensis *is smaller and lighter than *L. dorsalis*. The tibia of *L. sahamalazensis *seems to be longer than in *L. dorsalis*, although tarsus length does not differ.

#### Etymology

*L. sahamalazensis *is named after the type locality, the Sahamalaza Peninsula.

#### Distribution

The species is restricted to the type locality of the Sahamalaza Peninsula, with the Sambirano River most likely as its northern boundary. Further field studies are required to determine the exact distribution range.

Based on our analyses, the classification of sportive lemurs is now as in additional file 8.

## Conclusion

The combination of cytogenetic and molecular approaches reported here revealed important information about the diversity and evolution of the sportive lemurs. Phylogenetic relationships obtained from the mitochondrial sequence data are completely resolved and significantly supported, so that they most likely display the real evolutionary relationships among the analysed species and populations. Both methods were able to confirm the species status of the eight traditionally recognized species, while mitochondrial sequencing indicated in addition further cryptic speciation events in *L. dorsalis *and *L. ruficaudatus*. Especially in the light of less pronounced differences in pelage colouration and other external characteristics in nocturnal species compared to diurnal taxa, molecular studies are imperative. However, no chromosomal differences were detected between the three new proposed species and their respective sister taxa. Moreover, only a relatively small number of specimens per locality and only one mitochondrial locus were analysed in this study, so that additional (genetic) data, e.g. from nuclear DNA loci, are required to confirm the distinct species status of the populations. Furthermore, behavioural and morphological studies will provide independent data to delimit the species status of the three new forms. The discovery of three new possible primate species however, shows how diverse Madagascar's fauna is and how limited our knowledge currently still is. Ongoing work in the field and in the laboratory is urgently required to describe the complete diversity of sportive lemurs, especially of the different populations representing *L. leucopus, L. microdon *and *L. mustelinus*, and of other Malagasy lemurs. Greater knowledge of ecological, evolutionary and biogeographic patterns and processes will provide the necessary basis for protecting Madagascar's unique biodiversity.

## Methods

### Fieldwork

Samples from 68 individuals representing all currently recognized *Lepilemur *species were collected during field surveys in Madagascar (Fig. 1). Skin biopsies were taken under general anaesthesia with a 2 mg/kg injection of ketamine solution (Ketalar^® ^Parke-Davis) or GM2 [34]. A part of each sample was directly frozen in liquid nitrogen, while the other part was preserved with a cryoprotector (DMSO 10%) with the aim of growing fibroblast cultures. Other tissue samples were stored in 75% ethanol, or in Queen's lysis buffer [35]. From some animals, 0.5 ml blood was also taken in heparinized tubes for further lymphoblast cultures. From most animals, standard morphometric measurements, including body mass, head length and width, head-body length, tail length and hind foot length, were collected according to [36]. For morphometric comparisons, we included only individuals with a body mass of at least 660 g, because this is the lower limit of adult body mass for *L. ruficaudatus *in the Kirindy forest. However, morphometric data were collected by different people under various conditions, and hence we expect low inter-observer reliability. We therefore did not use morphometric data for species delimitation, but rather use them as a tool to present a picture of the taxa. Immediately after recovery from anaesthesia, animals were released in their respective capture areas.

### Cytogenetics

A total of 99 sportive lemur individuals with at least one specimen per locality was cytogenetically analysed during studies performed from 1975 till 2005 [8,9,26,37-39]. Chromosomes were prepared either from lymphoblast or fibroblast cultures, following classical methods [8,9,26,37-39]. Giemsa staining was performed for all 99 individuals. With the exception of one population, R-banded and C-banded chromosomes were prepared for at least one individual (see additional file 2).

### Molecular genetics

DNA from the biopsies was extracted using a standard proteinase K digestion, followed by a phenol chloroform extraction [40] with minor modifications [41], or isolated with the QIAamp DNA Mini Kit as recommended by the supplier.

The complete mitochondrial cytochrome b gene was amplified via PCR using the oligonucleotide primers CYT-LEP-L: 5'-AATGATATGAAAAACCATCGTTGTA-3' and CYT-LEP-H: 5'-GGCTTACAAGGCCGGGGTAA-3'. Standard, wax-mediated hot-start PCRs were carried out for 40 cycles, each with a denaturation step at 94°C for 60 s, annealing at 60°C for 60 s, and extension at 72°C for 90 s, followed by a final extension step at 72°C for 5 min. Aliquots of the PCR amplifications were checked by agarose gel electrophoresis. Subsequently, PCR products were cleaned using the Qiagen PCR Purification Kit and sequenced on an ABI 3100-Avant sequencer using the BigDye Terminator Cycle Sequencing Kit (Applied Biosystems), primers as indicated above and the internal primers CYT-LEP-L400: 5'-TGAGGACAAATATCATTCTGAGG-3' and CYT-LEP-H545: 5'-TGGAGTGCGAAGAATCGGGT-3'. The respective sequences were deposited in GenBank and are available under the accession numbers DQ108990-DQ109034 and DQ234881-DQ234900.

Sequences were easily aligned by eye due to the lack of insertions or deletions, and were checked for their potential to be correctly transcribed in order to eliminate data set contaminations with pseudogenes. For a comprehensive evaluation of the sequence data, we expanded our data set with orthologous sequences, already deposited at GenBank, from two *L. ruficaudatus *and one *L. dorsalis*. As outgroup for phylogenetic tree reconstructions, we selected *Phaner furcifer *because it displays the most similar orthologous sequence of all Malagasy lemurs to *Lepilemur *[29]. Further details about analysed individuals and sequences are summarized in Figure 1 and additional file 1.

Uncorrected pairwise differences within and between species and major populations were calculated with PAUP 4.0b10 [42] and DnaSP 3.52 [43].

A population aggregation analysis (PAA) was performed according to the diagnostic character framework described in [44]. Accordingly, fixed nucleotide characters provide the unit for which aggregation of taxonomic units occurs. For diagnosis, attributes whose fixed unique states unite a group (populations, species), to the exclusion of other groups, are considered characters. Polymorphic attributes, or traits, are indicative of population frequency differences. To identify diagnostic sites, sequences were imported into MacClade 3.0 [45].

Phylogenetic tree reconstructions were carried out with the maximum-parsimony (MP), neighbor-joining (NJ) and maximum-likelihood (ML) algorithms as implemented in PAUP or TREEPUZZLE 5.0 [46]. For MP analyses, all characters were treated as unordered and equally weighted throughout. A heuristic search was performed with the maximum number of trees set to 100. NJ and ML trees were constructed with the TVM + I (= 0.5156) + Γ (= 2.2387) model of sequence evolution as it was selected as best-fitting model with MODELTEST 3.06 [47], as well as with standard models. Relative support of internal nodes was performed by bootstrap analyses with 1,000 replications (MP, NJ), or by the quartet puzzling support values on the basis of 10,000 puzzling steps (ML).

## Authors' contributions

NA participated in the design of the study and the concept of the revision work on *Lepilemur*. She did the field work for *L. microdon*, *L. mustelinus*, *L. edwardsi *and *L. aeeclis*, took morphometric measurements and did a part of the cytogenetic study in the Institut Pasteur de Madagascar (IPM) in Antananarivo.

JLF designed *Lepilemur *specific primers, generated several sequences, performed the statistical analysis of the phylogenetic data and wrote a draft version of the MS in collaboration with YR.

ChR designed *Lepilemur *specific primers, generated several sequences, performed the statistical analysis of the phylogenetic data and wrote the draft and final version of the MS in collaboration with DZ.

DZ organised, lead and conducted the expedition to collect data and samples of *L. ruficaudatus *south of the Morondava River. He also sampled *L. ruficaudatus *in the Kirindy Forest and took morphometric measurements in collaboration with RH. DZ generated sequences from these individuals, did the statistical analysis of the morphometric data and wrote the draft and final version of the MS in collaboration with ChR.

UT conceived, planned, organised, lead and conducted the expeditions to collect the samples, data and pictures of *L. aeeclis *sp. nov. in the field, participated in the design and drafting of the MS, critically revised it several times and approved the final version.

ClR participated in the design of the study and the concept of the revision work on *Lepilemur*, in collaboration with YR and NA. He organized and conceptualized the field work of NA, participated in the collection of samples of *L. edwardsi *and participated in a revision of a draft version of the MS.

IR participated in the design of the study and the concept of the revision work on *Lepilemur*. She did the field work for *L. septentrionalis*, *L. ankaranensis*, *L. dorsalis *in Ambanja and part of Nosy Be.

JG sampled *L. randrianasoli *and *L. ruficaudatus *and provided morphometric data and tissue samples.

BM organized financed and conceptualized part of the field work on on Nosy Be (with CL), in Sahamalaza Peninsula (with TH and IR), on the ridge of Andrafiamena (with IR) and in Ankarana (with IR). At several of these sites, he collected samples and animal measurements.

RH sampled *L. ruficaudatus *south of the Tsiribihina River and took morphometric measurements in collaboration with DZ and he took photographs of L. sahamalazensis sp. nov. in the field.

LW designed *Lepilemur *specific primers and generated several sequences.

AZ participated in the expeditions to collect data on *L. aeeclis *sp. nov., and found the skull of the animal of the type series. He considerably facilitated field work and data collection.

CL did field work on Nosy Be.

TH did field work on Sahamalaza Peninsula.

EZ organized, financed and conceptualized part of the field work and revised the MS.

UR organized, financed and conceptualized part of the field work and revised the MS.

MC conducted part of the field work including capturing, measuring and sampling the Sahamalaza population, and revised the MS.

JT participated in the design of the study and the concept of the revision work on *Lepilemur*.

IT provided systematic input and checked the MS.

YR participated in the design of the study and the concept of the revision work on *Lepilemur*, wrote a draft version of the MS, critically revised it several times and approved its final version.

## Supplementary Material

Additional File 1 A table showing details on studied sportive lemur individuals. Click here for file

Additional File 2 A table showing diploid number (2N) and chromosomal rearrangements among species and populations [9]. Click here for file

Additional File 3 A table showing minimum and maximum uncorrected pairwise genetic differences (in %) within and among analysed species and populations based on complete mitochondrial cytochrome b sequence data. Click here for file

Additional File 4 A table showing number of diagnostic characters as obtained from population aggregation analysis (PAA). Click here for file

Additional File 5 A table showing morphometric measurements for the *L. aeeclis *syntype skull Click here for file

Additional File 6 A table showing morphometric measurements of *L. randrianasoli *and *L. aeeclis *in comparison with *L. ruficaudatus*. Click here for file

Additional File 7 A table showing morphometric measurements for *L. sahamalazensis *in comparison to *L. dorsalis*. Click here for file

Additional File 8 A table showing classification of sportive lemurs. Click here for file
