# Supplementary material for: Molecular phylogeny and taxonomic revision of the sportive lemurs (Lepilemur, Primates)
Source: BMC Evol Biol. 2006 Feb 23;6:17. doi: 10.1186/1471-2148-6-17 (PMC1397877; doi:10.1186/1471-2148-6-17)

**Table 1:** Details on studied sportive lemur individuals

| **species** | **Areas of capture** | **coordinates** | **abbreviations (GenBank)** | **karyograms*** |
| --- | --- | --- | --- | --- |
| *Phaner furcifer* | Kirindy (CFPF) | 44°39'E, 20°04'S | - (AY441456) | - |
| *L. leucopus* | - | - | Lle1 (DQ109007) | 5 (1) |
| *L. ruficaudatus* | Manamby-sud | 44°49'E, 20°28'S | Lru1 (DQ109012),  Lru20 (DQ234895),  Lru21 (DQ234896),  Lru25 (DQ234897),  Lru26 (DQ234898) | 3 (1) |
| *L. ruficaudatus* | Kirindy (CFPF) | 44°39'E, 20°04'S | Lru2 (DQ109013),  Lru3 (DQ109014),  Lru4 (DQ109015),  Lru5 (DQ109017) | 2 (1) |
| *L. ruficaudatus* | Kiboy | 44°40’E, 19°47’S | Lru6 (DQ109018) | - |
| *L. ruficaudatus* | Antevado | 44°03’E, 20°52’S | Lru7 (DQ109016) | - |
| *L. ruficaudatus* | - | - | Lru8 (DQ109011) | - |
| *L. ruficaudatus* | Andramasay | 44°29'E, 19°28'S | Lru9 (AY441463),  Lru10 (DQ109019),  Lru19 (DQ234891),  Lru22 (DQ234892),  Lru23 (DQ234893),  Lru24 (DQ234894) | 2 (2) |
| *L. ruficaudatus* | Ambalarano | 44°46’E, 19°00’S | Lru17 (DQ234890) |  |
| *L. ruficaudatus* | - | - | Lru11 (AY321456) | - |
| *L. ruficaudatus* | Antafia - Anjahamena | 45°54'E, 16°03'S | Lru12 (DQ108999),  Lru13 (DQ109000),  Lru14 (DQ109001),  Lru15 (DQ109002),  Lru16 (DQ109003)  Lru18 (DQ234899) | 1 |
| *L. edwardsi* | Ampijoroa | 46°49'E, 16°19'S | Led1 (DQ109006) | 5 (1) |
| *L. edwardsi* | Andofombombe | 46°55’E, 16°19’S | Led2 (DQ109004),  Led3 (DQ109005),  Led4 (DQ234888) | - |
| *L. microdon* | Vohiparara | 47°24’E, 21°14’S | Lmi1 (DQ109008) | 1 (1) |
| *L. microdon* | Antarando | 47°25’E, 21°14’S | Lmi2 (DQ109009),  Lmi3 (DQ109010) | 1 (1) |
| *L. microdon* | Ambatolampy | 47°14’E, 19°51’S | Lmi4 (DQ234889) |  |
| *L. dorsalis* | Nosy Be | 48°20’E, 13°23’S | Ldo1 (AY441464),  Ldo2 (DQ108993),  Ldo3 (DQ108994),  Ldo4 (DQ108998),  Ldo13 (DQ234885) | 13 (1) |
| *L. dorsalis* | Ambanja | 48°35’E, 13°28’S | Ldo5 (DQ108995),  Ldo6 (DQ108996),  Ldo7 (DQ108997),  Ldo14 (DQ234886),  Ldo15 (DQ234887) | 2 (1) |
| *L. dorsalis* | Sahamalaza | 47°58’E, 14°16’S | Ldo8 (DQ108990),  Ldo9 (DQ108991),  Ldo10 (DQ108992),  Ldo11 (DQ234882),  Ldo12 (DQ234883) | 3 (2) |
| *L. ankaranensis* | Ankarana | 49°09'E, 12°50'S | Lan1 (DQ109028),  Lan2 (DQ109029),  Lan3 (DQ109030),  Lan4 (DQ109031),  Lan5 (DQ109032) | 12 (1) |
| *L. ankaranensis* | Analamera | 49°30’E, 12°50’S | Lan6 (DQ109022),  Lan7 (DQ109023),  Lan8 (DQ109024),  Lan12 (DQ234884) | 12 (1) |
| *L. ankaranensis* | Andrafiamena | 49°20’E, 12°55’S | Lan9 (DQ109025),  Lan10 (DQ109027),  Lan13 (DQ234881) | 7 (1) |
| *L. ankaranensis* | - | - | Lan11 (DQ109026) | - |
| *L. septentrionalis* | Sahafary | 49°25’E, 12°38’S | Lse1 (DQ109020),  Lse2 (DQ109021),  Lse3 (DQ234900) | 28 (3) |
| *L. mustelinus* | Behasina | 47°51’E, 19°44’S | Lmu1 (DQ109033) | 1 (1) |
| *L. mustelinus* | near Mantadia | 48°25’E, 18°47’S | Lmu2 (DQ109034) | 1 (1) |

* number of individuals karyotyped from 1975 till 2005, in brackets with R-banding

**Table 2:** Diploid number (2N) and chromosomal rearrangements among species and populations

|  | 2N | LruNT | LruST | LruSB | Led | Lmu | LdoAN | LdoS | Lse | Lan | Lle | Lmi | n |
| --- | --- | --- | --- | --- | --- | --- | --- | --- | --- | --- | --- | --- | --- |
| LruNT | 20 | - |  |  |  |  |  |  |  |  |  |  | 2 (2) |
| LruST | 20 | 0 | - |  |  |  |  |  |  |  |  |  | 5 (2) |
| LruSB | 20 | 0 | 0 | - |  |  |  |  |  |  |  |  | 1 |
| Led | 22 | 12 | 12 | 12 | - |  |  |  |  |  |  |  | 5 (1) |
| Lmu | 34 | 17 | 17 | 17 | 19 | - |  |  |  |  |  |  | 2 (2) |
| LdoAN | 26 | 10 | 10 | 10 | 8 | 15 | - |  |  |  |  |  | 15 (2) |
| LdoS | 26 | 10 | 10 | 10 | 8 | 15 | 0 | - |  |  |  |  | 3 (2) |
| Lse | 34/36 | 11 | 11 | 11 | 13 | 14 | 9 | 9 | - |  |  |  | 28 (3) |
| Lan | 36/38 | 12 | 12 | 12 | 14 | 15 | 10 | 10 | 1 | - |  |  | 31 (2) |
| Lle | 26 | 12 | 12 | 12 | 12 | 17 | 6 | 6 | 11 | 12 | - |  | 5 (1) |
| Lmi | 24 | 11 | 11 | 11 | 3 | 18 | 7 | 7 | 12 | 13 | 11 | - | 2 (2) |
| Total |  |  |  |  |  |  |  |  |  |  |  |  | 99 (19) |

Abbreviations are: LruNT = *L. ruficaudatus* (north of Tsiribihina); LruST = *L. ruficaudatus* (south of Tsiribihina); LruSB = *L. ruficaudatus* (south of Betsiboka); Led = *L. edwardsi*; Lmu = *L. mustelinus*; LdoAN = *L. dorsalis* (Ambanja/ Nosy Be); LdoS = *L. dorsalis* (Sahamalaza Peninsula); Lse = *L. septentrionalis*; Lan = *L. ankaranensis*; Lle = *L. leucopus*; Lmi = *L.* *microdon*; n = number of individuals karyotyped from 1975 till 2005, in brackets with R-banding.

**Table 3:** Minimum and maximum uncorrected pairwise genetic differences (in %) within and among analysed species and populations based on complete mitochondrial cytochrome b sequence data

|  | **1** | **2** | **3** | **4** | **5** | **6** | **7** | **8** | **9** | **10** | **11** |
| --- | --- | --- | --- | --- | --- | --- | --- | --- | --- | --- | --- |
| **1 *L. leucopus*** | - |  |  |  |  |  |  |  |  |  |  |
| **2 *L. ruficaudatus***  (south of Tsiribihina) | 10.26-10.70 | 0.00-1.84 |  |  |  |  |  |  |  |  |  |
| **3 *L. ruficaudatus***  (north of Tsiribihina) | 10.44-10.70 | 6.15-7.11 | 0.00-2.28 |  |  |  |  |  |  |  |  |
| **4 *L. ruficaudatus***  (south of Betsiboka) | 11.23-11.32 | 7.11-7.63 | 5.88-6.75 | 0.00-0.26 |  |  |  |  |  |  |  |
| **5 *L. edwardsi*** | 12.54-12.63 | 12.46-12.98 | 11.75-12.02 | 12.46-12.63 | 0.00-0.09 |  |  |  |  |  |  |
| **6 *L. microdon*** | 11.40 | 11.75-12.46 | 11.32-11.84 | 12.28-12.72 | 9.21-9.47 | 0.00-0.53 |  |  |  |  |  |
| **7 *L. dorsalis***  (Sahamalaza Peninsula) | 13.07-13.42 | 11.23-12.02 | 11.58-12.28 | 12.46-13.07 | 12.02-12.54 | 10.97-11.40 | 0.00-1.05 |  |  |  |  |
| **8 *L. dorsalis***  (Ambanja/Nosy Be) | 12.63-12.72 | 10.97-11.93 | 11.49-11.67 | 10.97-11.32 | 10.70-10.79 | 10.26-10.53 | 5.18-5.88 | 0.00-0.26 |  |  |  |
| ***9 L. ankaranensis*** | 12.54-12.72 | 10.44-11.14 | 10.88-11.75 | 10.97-11.32 | 10.44-11.14 | 10.00-10.40 | 4.56-5.35 | 2.90-3.60 | 0.00-1.58 |  |  |
| **10 *L. septentrionalis*** | 13.16-13.86 | 11.14-12.72 | 10.79-12.02 | 12.46-12.63 | 10.70-11.58 | 9.83-10.79 | 9.30-11.14 | 7.46-8.95 | 7.37-9.39 | 0.00-1.58 |  |
| **11 *L. mustelinus*** | 15.44 | 14.47-15.35 | 14.82-15.61 | 15.61-15.97 | 16.23-16.58 | 16.14-16.40 | 16.40-16.82 | 16.05-16.23 | 15.35-15.70 | 15.88-16.49 | 0.61 |

**Table 4:** Number of diagnostic characters as obtained from population aggregation analysis (PAA)

|  | Lle | LruST | LruNT | LruSB | Led | Lmi | LdoS | LdoAN | Lan | Lse | Lmu |
| --- | --- | --- | --- | --- | --- | --- | --- | --- | --- | --- | --- |
| Lle | - |  |  |  |  |  |  |  |  |  |  |
| LruST | 115 | - |  |  |  |  |  |  |  |  |  |
| LruNT | 115 | 68 | - |  |  |  |  |  |  |  |  |
| LruSB | 124 | 78 | 64 | - |  |  |  |  |  |  |  |
| Led | 139 | 140 | 131 | 138 | - |  |  |  |  |  |  |
| Lmi | 125 | 131 | 125 | 135 | 101 | - |  |  |  |  |  |
| LdoS | 146 | 126 | 130 | 138 | 134 | 121 | - |  |  |  |  |
| LdoAN | 141 | 124 | 128 | 122 | 119 | 114 | 57 | - |  |  |  |
| Lan | 142 | 117 | 123 | 123 | 115 | 111 | 50 | 32 | - |  |  |
| Lse | 145 | 124 | 119 | 130 | 116 | 107 | 102 | 81 | 82 | - |  |
| Lmu | 171 | 163 | 165 | 174 | 183 | 179 | 183 | 179 | 172 | 173 | - |

Abbreviations are: Lle = *L. leucopus*; LruST = *L. ruficaudatus* (south of Tsiribihina); LruNT = *L. ruficaudatus* (north of Tsiribihina); LruSB = *L. ruficaudatus* (south of Betsiboka); Led = *L. edwardsi*; Lmi = *L.* *microdon*; LdoS = *L. dorsalis* (Sahamalaza Peninsula); LdoAN = *L. dorsalis* (Ambanja/Nosy Be); Lan = *L. ankaranensis*; Lse = *L. septentrionalis*; Lmu = *L. mustelinus*.

**Table 5:** Morphometric measurements for the *L. aeeclis* syntype skull

| **Skull (syntype UM 2003-Lem-100)** | | | **mm** |
| --- | --- | --- | --- |
| skull length | | | 59.0 |
| zygomatic width | | | 40.0 |
| bicanine width | | | 14.5 |
| facial length | | | 29.5 |
| maxillary toothrow | | | 21.8 |
| mandibular toothrow | | | 24.1 |
| orbital diameter min (average left and right) | | | 14.3 |
| orbital diameter max (average left and right) | | | 15.1 |
| postorbital width | | | 20.4 |
| **mandibel** | | | **mm** |
| hight of ramus mandibularis (average left and right) | | | 23.7 |
| length of mandible (average left and right) | | | 45.4 |
| **dentition** | | | **mm** |
| tooth comb length | | | 6.3 |
| tooth comb basal width | | | 4.7 |
| tooth apical width | | | 3.9 |
| premolar tooth row length | | | 10.8 |
| molar tooth row length | | | 13.0 |
| upper dentition LxW | mm | lower dentition LxW | mm |
| C | 4.7 x 2.2 |  |  |
| P2 | 3.7 x 2.2 | P2 | 4.5 x 2.2 |
| P3 | 3.5 x 3.0 | P3 | 3.8 x 2.2 |
| P4 | 3.2 x 3.4 | P4 | 3.7 x 2.2 |
| M1 | 4.3 x 4.3 | M1 | 4.3 x 2.9 |
| M2 | 4.4 x 4.5 | M2 | 4.2 x 3.0 |
| M3 | 3.7 x 3.9 | M3 | 4.6 x 2.7 |

**Table 6:** Morphometric measurements of *L. randrianasoli* and *L. aeeclis* in comparison with *L. ruficaudatus*

|  | ***L. randrianasoli* (n=9)**  **Locality: Andramasay** | | ***L. ruficaudatus* (n=45)**  **Locality: Kirindy/CFPF** | | ***L. aeeclis* (n=5)**  **Locality: Antafia/Anjahamena** | |
| --- | --- | --- | --- | --- | --- | --- |
|  | **female (n=4)** | **male (n=5)** | **female (n= 24)** | **male (n=21)** | **female (n=2)** | **male (n=3)** |
| ^1^ body mass (g)** | 793 (740 - 880) | 717 (660 - 760) | 803 (670 - 930) | 806 (712 - 896) | 909 (845 - 972) | 868 (763 - 940) |
| ^2^ head-body length (mm) |  |  | 309 (289 - 335) | 308 (287 - 344) | 300 (295 - 305) | 303 (285 - 315) |
| ^3^ head length (mm) | 63.6 (60.8 - 66.2) | 63.9 (63.1 - 66.0) | 62.2 (57.8 - 65.3) | 62.8 (60.4 - 65.8) | 63.0 (62.0 - 64.0) | 63.0 (60.0 - 65.0) |
| ^4^ head width (mm)** | 37.4 (36.5 - 38.8) | 38.0 (37.3 - 38.8) | 39.2 (37.2 - 42.0) | 39.7 (37.3 - 43.0) | 39.0  (n=1) | 43.3 (37.0 - 48.0) |
| ^5^ lower hind leg length (mm) | 102 (100 - 105) | 101 (97 - 107) |  |  | 101 (100 - 102) | 99 (94 - 105) |
| ^6^ hind foot length (mm)*** | 78.8 (78 - 80) | 78.2 (74 - 81) | 68.7 (63 - 74) | 68.2 (63 - 74) | 79.0 (79 - 79) | 77.7 (75 .- 80) |
| ^7^ tail length (mm) | 240 (220 - 253) | 226 (209 - 244)  (n=4) | 249 (203 - 281) | 238 (209 - 270)  (n=20) | 255 (250 - 260) | 252 (240 - 260) |

^1^ANOVA body mass: site F_2,53_ = 7.18, p<0.01; sex:F_1,53_ = 2.45, ns; site x sex F_2,53_ = 1.63, ns

^2^ANOVA head-body length: site F_1,46_ = 1.19, ns; sex:F_1,46_ = 0.03, ns; site x sex F_1,46_ = 0.11, ns

^3^ANOVA head length: site F_2,53_ = 1.78, ns; sex:F_1,53_ = 0.21, ns; site x sex F_2,53_ = 0.08, ns

^4^ANOVA head width: site F_2,52_ = 6.19, p<0.01; sex:F_1,52_ = 6.06, p<0.05; site x sex F_2,52_ = 1.83, ns

^5^ANOVA lower hind leg length: site F_1,10_ = 0.69, ns; sex:F_1,10_ = 0.82, ns; site x sex F_1,10_ = 0.03, ns

^6^ANOVA hind foot length: site F_2,53_ = 59.53, p<0.001; sex:F_1,53_ = 0.49, ns; site x sex F_2,53_ = 0.04, ns

^7^ANOVA tail length: site F_2,51_ = 2.27, ns; sex:F_1,51_ = 1.91, ns; site x sex F_2,51_ = 0.15, ns

**Table 7:** Morphometric measurements for *L. sahamalazensis* in comparison to *L. dorsalis*

|  | ***L. sahamalazensis* (n=6)**  **Locality: Ankarafa forest** | | ***L. dorsalis* (n=18)**  Locality: Nosy Be | |
| --- | --- | --- | --- | --- |
|  | **males (n=2)** | **females (n=4)** | **males (n=9)** | **females (n=9)** |
| ^1^body mass (g)* | 691 (687-694) | 787 (740 – 892) | 817 (700 – 900) | 923 (660 – 1110) |
| ^2^head-body length (mm)*** | 258 (252 – 264) | 259 (254 – 266) | 305 (250 – 340) | 319 (280 – 355) |
| head length (mm) | 56.5 (56.2 – 56.8) | 57.1 (55.6 – 58.4) |  |  |
| head width (mm) | 35.2 (34.6 – 35.8) | 35.1 (34.1 – 36.8) |  |  |
| ^3^tibia length (mm)*** | 103.0 (102 – 104) | 100.5 (98 – 105) | 86.4 (80 – 93) | 87.8 (81 – 102) |
| ^4^tarsus length (mm) | 48.2 (48 – 49) | 49.3 (47 – 52) | 48.2 (45 – 52) | 47.4 (45 – 51) |
| ^5^tail length (mm) | 260  (n=1) | 271 (267 – 274)  (n=2) | 257 (244 – 270)  (n=8) | 249 (230 – 270)  (n=8) |
| ^6^ear length (mm) | 25.4 (24 – 27) | 25.3 (23 – 28) | 22.6 (21 – 25) | 23.6 (19 – 31)  (n=8) |

^1^ANOVA body mass: site F_1,20_ = 5.91, p<0.05; sex:F_1,20_ = 3.51, p<0.08; site x sex F_1,20_ = 0.01, ns

^2^ANOVA head-body length: site F_1,20_ = 17.31, P<0.001; sex:F_1,20_ = 0.37, ns; site x sex F_1,20_ = 0.30, ns

^3^ANOVA tibia length: site F_1,20_ = 29.16, p<0.001; sex:F_1,20_ = 0.04, ns; site x sex F_1,20_ = 0.51, ns

^4^ANOVA tarsus length: site F_1,20_ = 0.70, ns; sex:F_1,20_ = 0.02, ns; site x sex F_1,20_ = 0.73, ns

^5^ANOVA tail length: site F_1,15_ = 2.13, ns; sex:F_1,15_ = 0.02, ns; site x sex F_1,15_ = 1.29, ns

^6^ANOVA tail length: site F_1,19_ = 3.10, p<0.10; sex:F_1,19_ = 0.11, ns; site x sex F_1,19_ = 0.17, ns

Table 8: Classification of sportive lemurs

| Family LEPILEMURIDAE | |
| --- | --- |
| Genus *Lepilemur* I. Geoffroy, 1851 | |
| Species | Distribution |
| *L. mustelinus*  I. Geoffroy, 1851 | East Madagascar, north of Andevoranto |
| *L. ruficaudatus*  A. Grandidier, 1867 | West Madagascar, south of Tsiribihina River,  possibly south to Onilahy River |
| *L. randrianasoli** sp nova | West Madagascar, north of Tsiribihina River,  northern range limit not yet determined |
| *L. aeeclis** sp. nova | West Madagascar, south of Betsiboka River,  southern range limit not yet determined |
| *L. dorsalis*  Gray, 1871 | North-West Madagascar |
| *L. sahamalazensis** sp nova | North-West Madagascar, Sahamalaza Peninsula, exact range limits not yet determined |
| *L. edwardsi*  Forsyth Major, 1894 | North-West Madagascar, Mahajanga region |
| *L. microdon*  Forsyth Major, 1894 | East Madagascar, south of Tamatave |
| *L. leucopus*  Forsyth Major, 1894 | South Madagascar, south of Onilahy River |
| *L. septentrionalis*  Rumpler and Albignac, 1975 | Far North of Madagascar |
| L. ankaranensis  Rumpler and Albignac, 1975 | North Madagascar, Ankarana Massif |

**Figure 1**

**Figure 2**


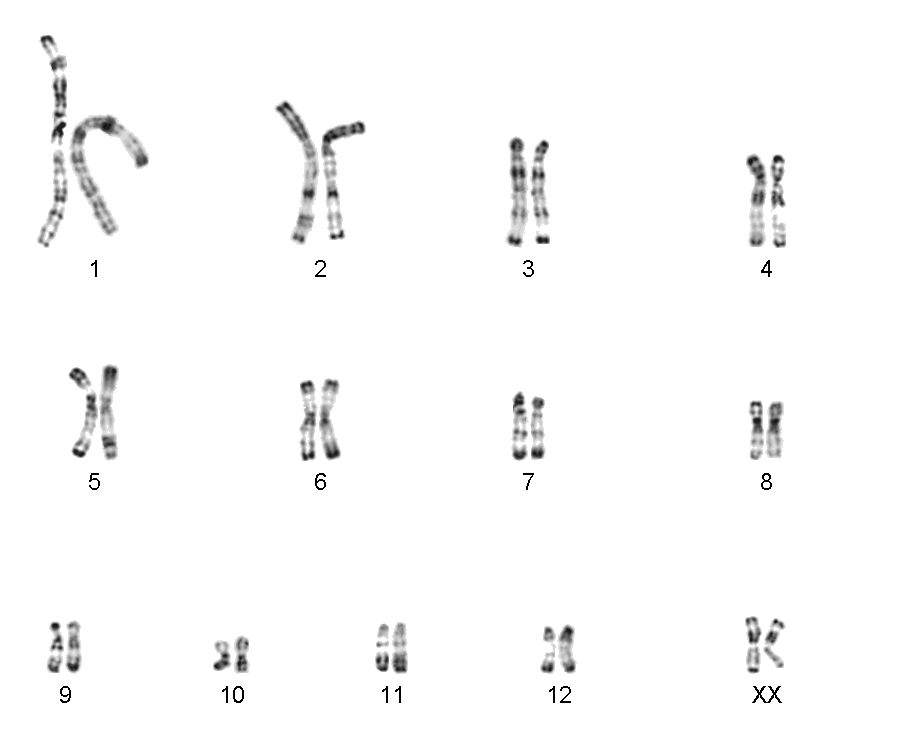


**Figure 3**


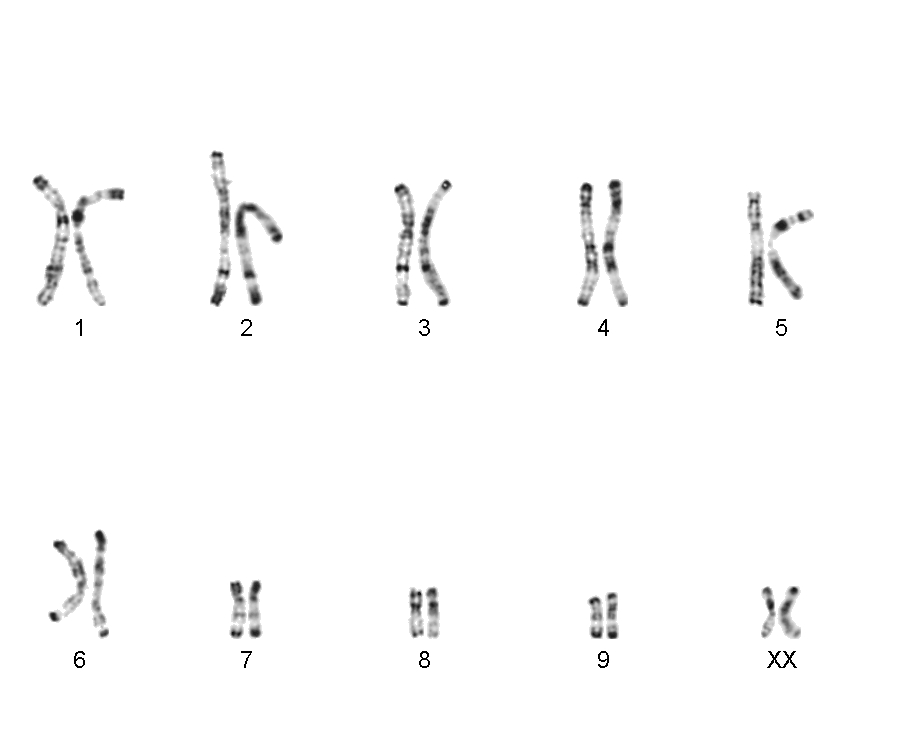


**Figure 4**


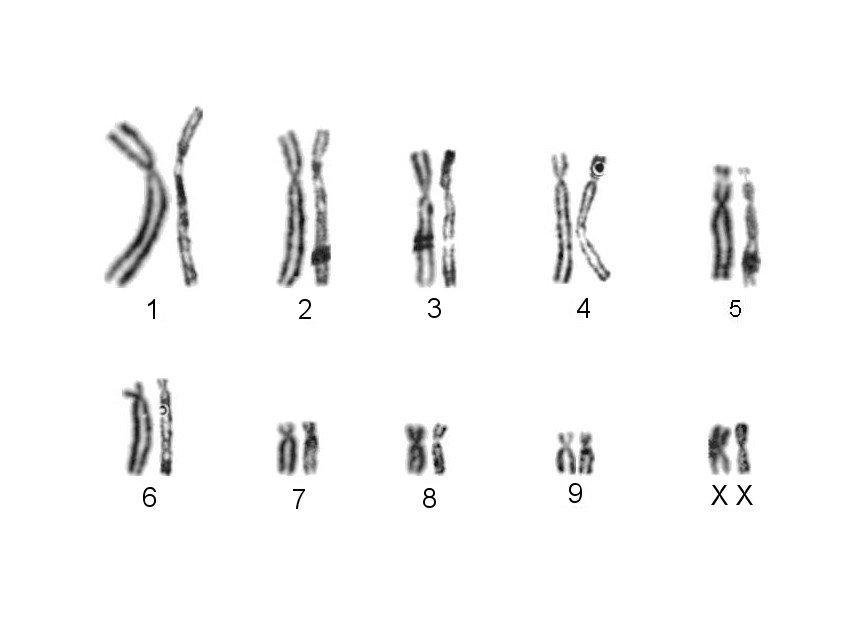


**Figure 5**

**Figure 6**

a) b)


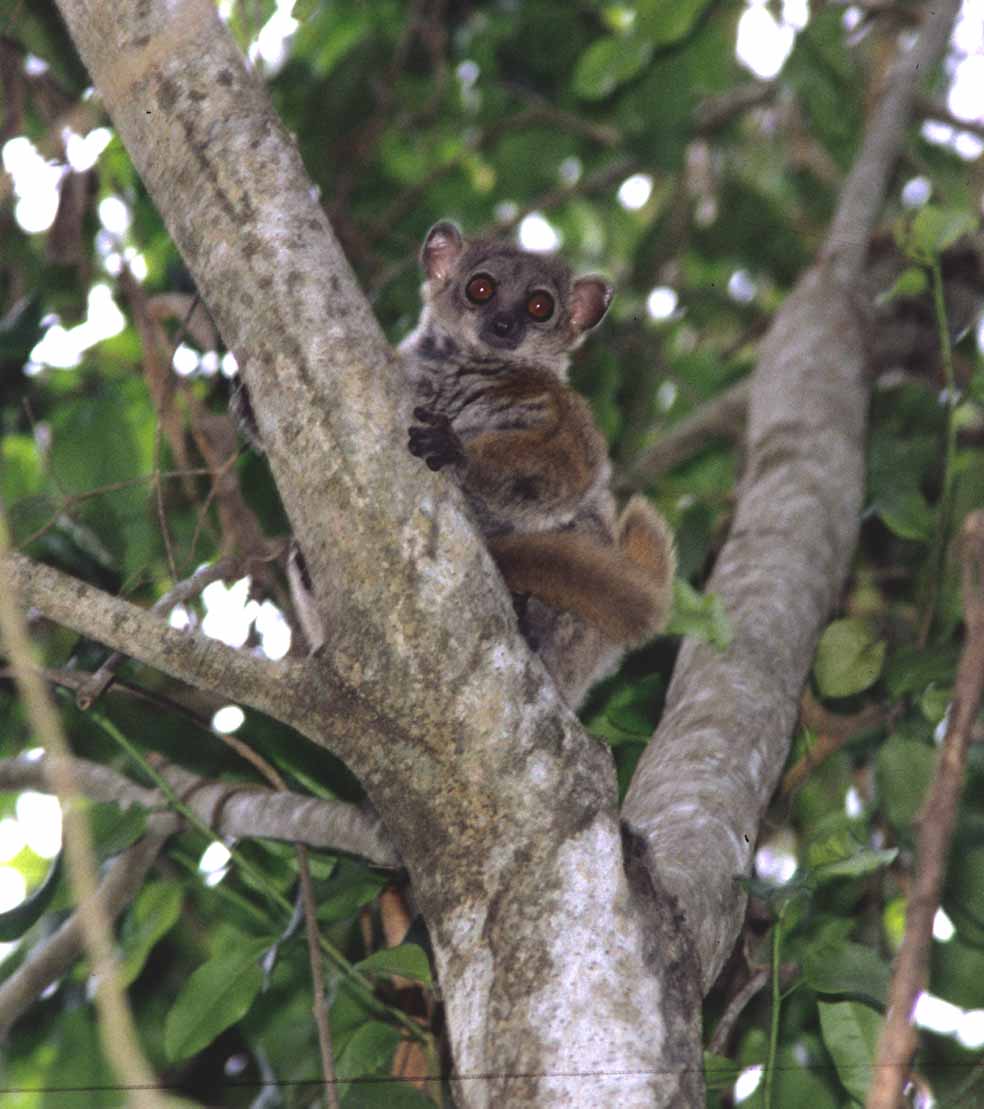
….
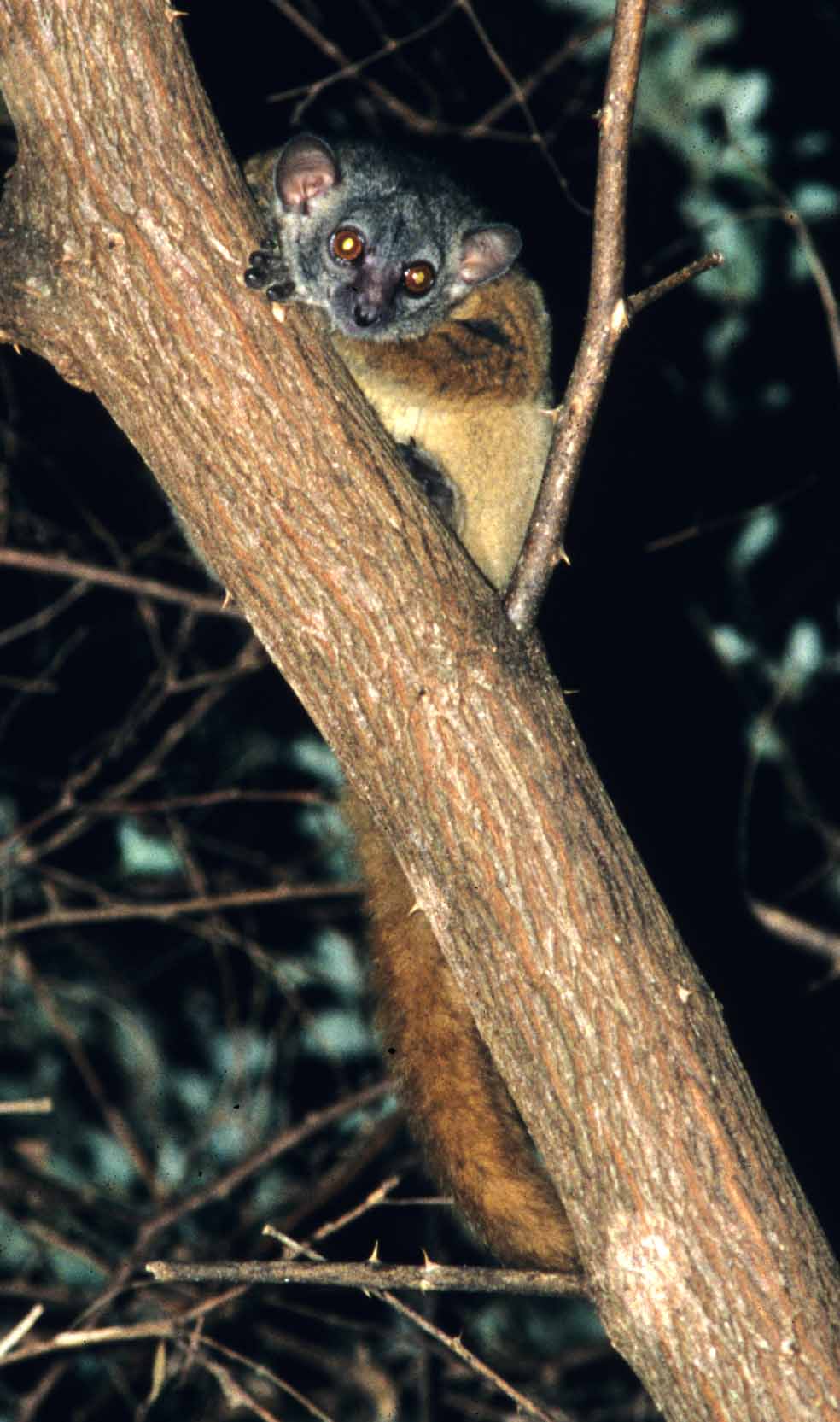


**Figure 7**

a) b)


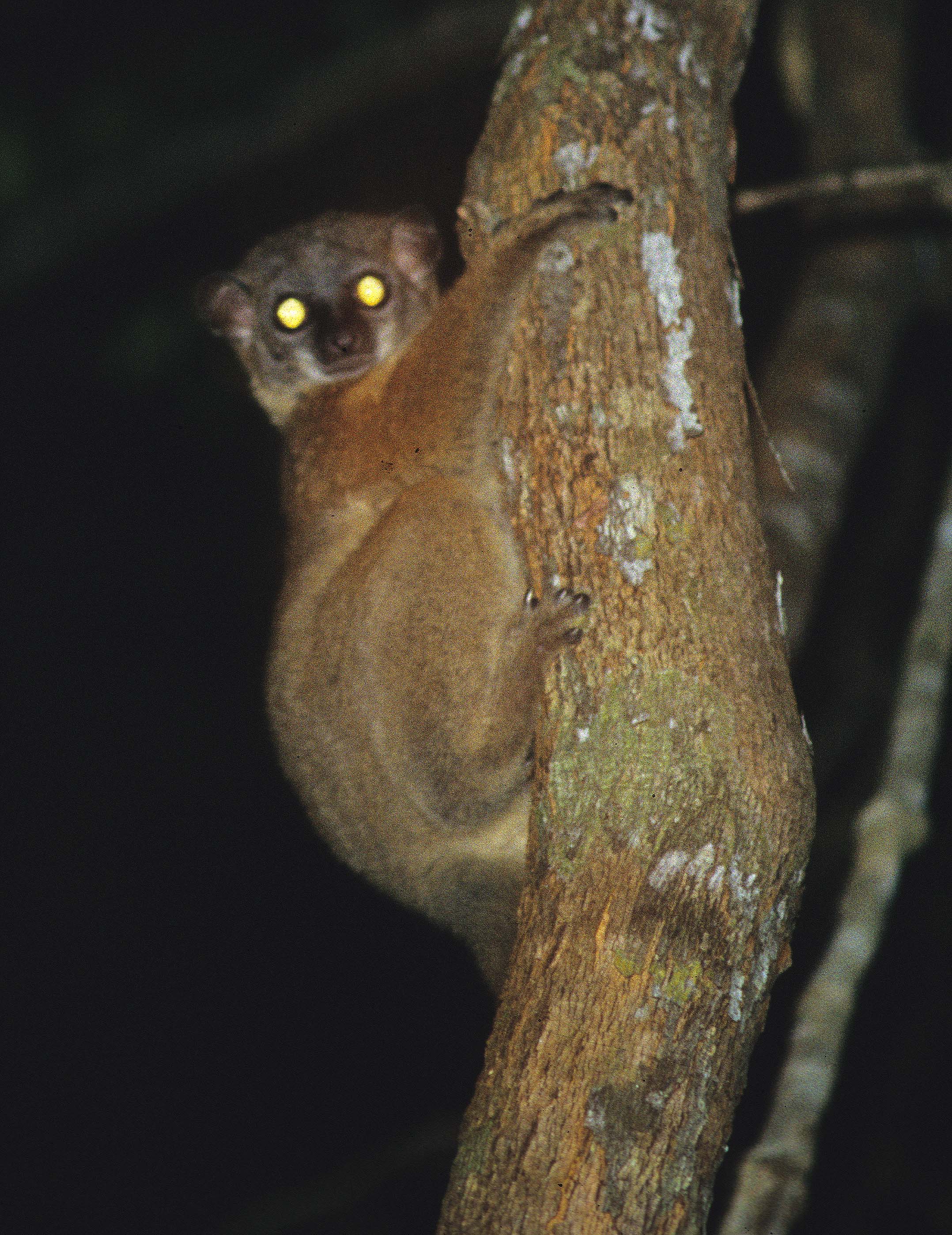

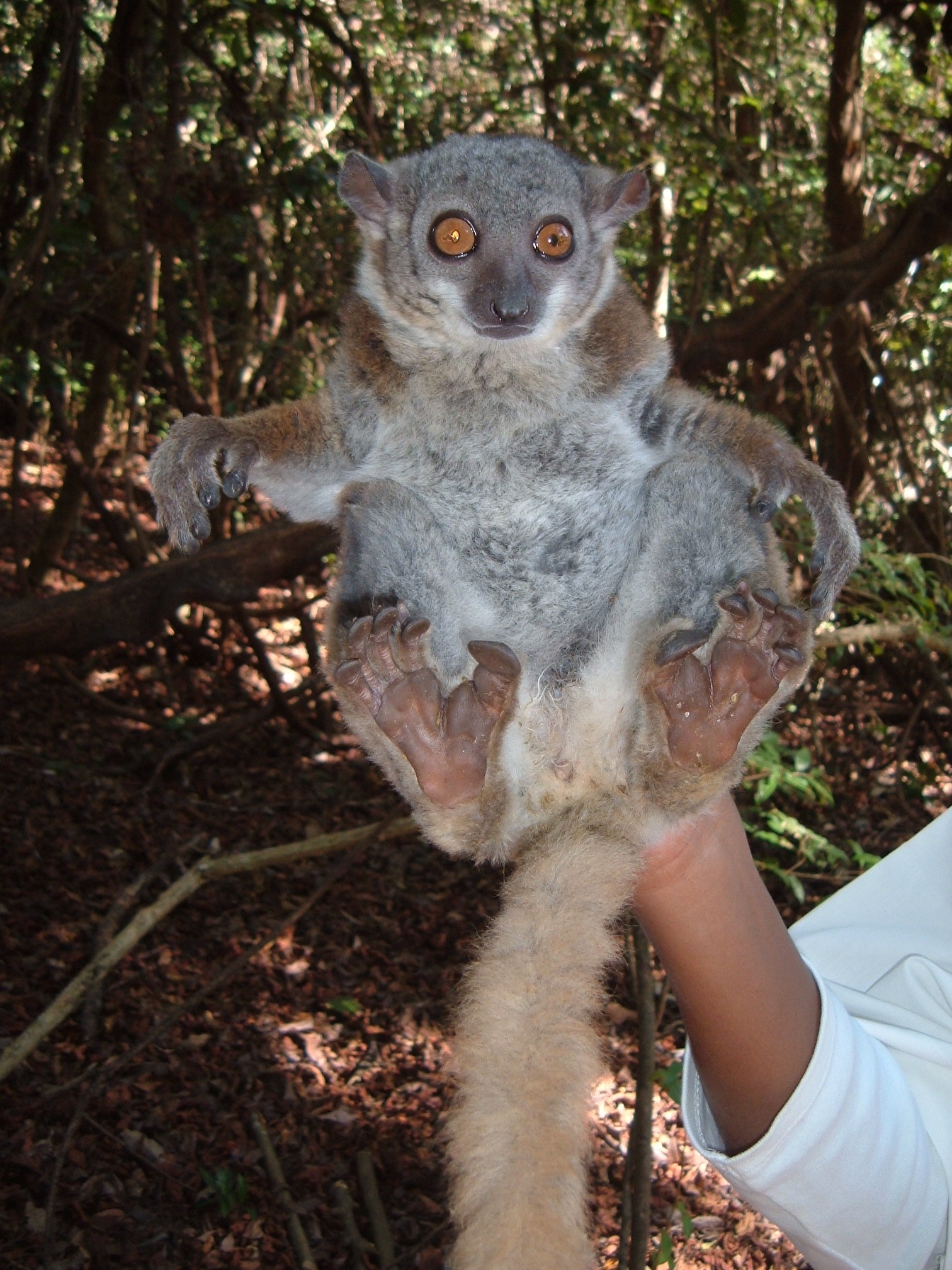


**Figure 8**

a) b)


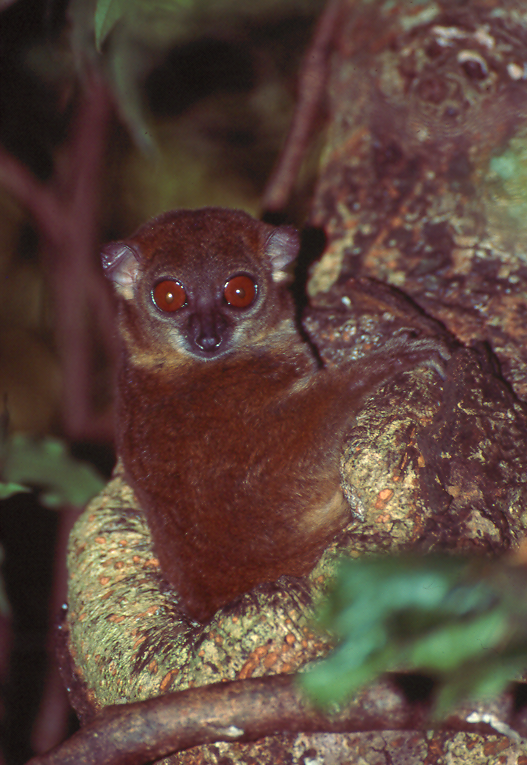

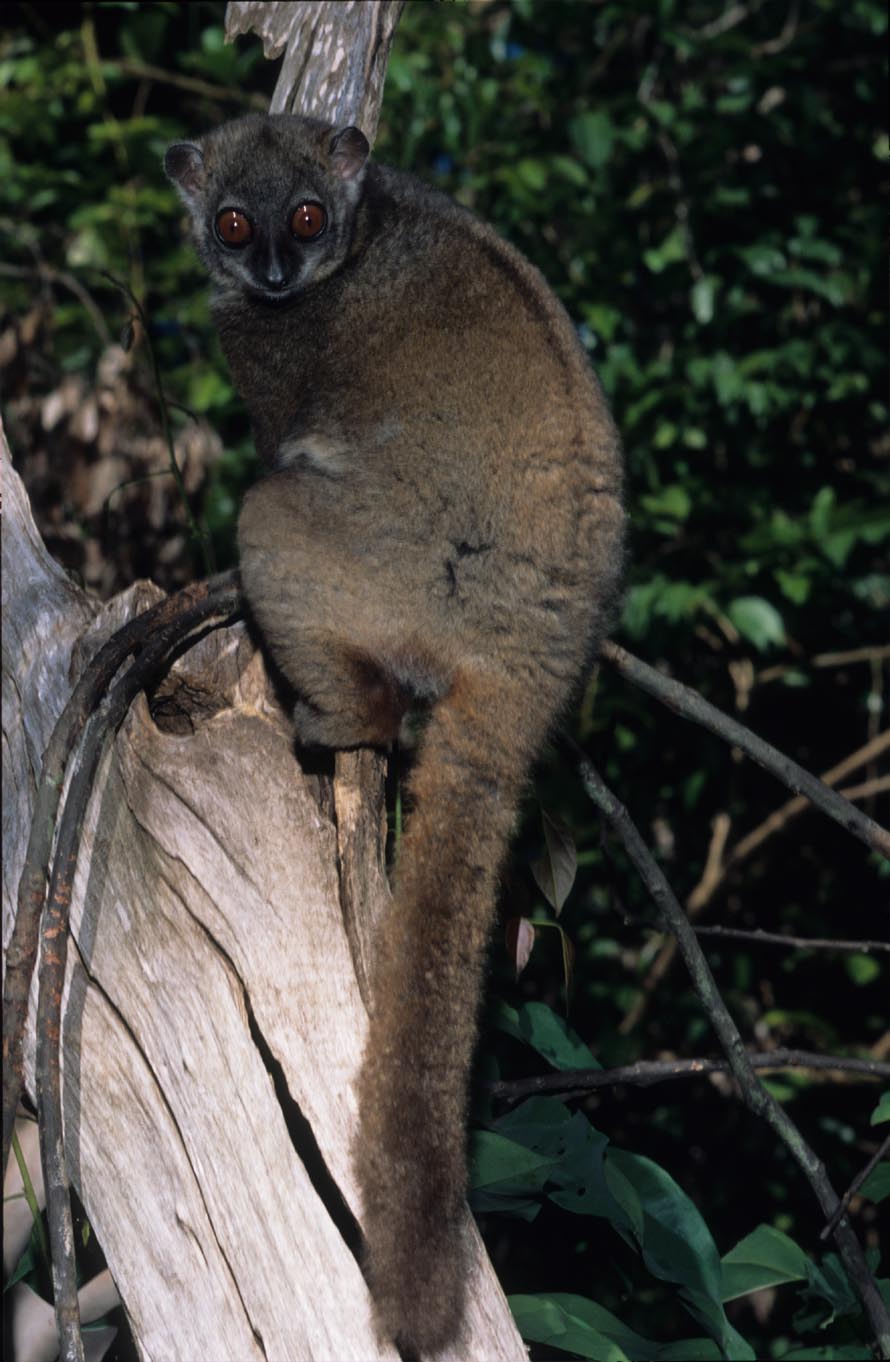

Supplement: Additional File 1 — A table showing details on studied sportive lemur individuals. [file 1471-2148-6-17-S1.doc]
