# Supplementary material for: Molecular phylogeny and taxonomic revision of the sportive lemurs (Lepilemur, Primates)
Source: BMC Evol Biol. 2006 Feb 23;6:17. doi: 10.1186/1471-2148-6-17 (PMC1397877; doi:10.1186/1471-2148-6-17)
Supplement: Additional File 2 — A table showing diploid number (2N) and chromosomal rearrangements among species and populations [9]. [file 1471-2148-6-17-S2.doc]

**Table 2:** Diploid number (2N) and chromosomal rearrangements among species and populations [9]

|  | 2N | LruNT | LruST | LruSB | Led | Lmu | LdoAN | LdoS | Lse | Lan | Lle | Lmi | n |
| --- | --- | --- | --- | --- | --- | --- | --- | --- | --- | --- | --- | --- | --- |
| LruNT | 20 | - |  |  |  |  |  |  |  |  |  |  | 2 (2) |
| LruST | 20 | 0 | - |  |  |  |  |  |  |  |  |  | 5 (2) |
| LruSB | 20 | 0 | 0 | - |  |  |  |  |  |  |  |  | 1 |
| Led | 22 | 12 | 12 | 12 | - |  |  |  |  |  |  |  | 5 (1) |
| Lmu | 34 | 17 | 17 | 17 | 19 | - |  |  |  |  |  |  | 2 (2) |
| LdoAN | 26 | 10 | 10 | 10 | 8 | 15 | - |  |  |  |  |  | 15 (2) |
| LdoS | 26 | 10 | 10 | 10 | 8 | 15 | 0 | - |  |  |  |  | 3 (2) |
| Lse | 34/36 | 11 | 11 | 11 | 13 | 14 | 9 | 9 | - |  |  |  | 28 (3) |
| Lan | 36/38 | 12 | 12 | 12 | 14 | 15 | 10 | 10 | 1 | - |  |  | 31 (2) |
| Lle | 26 | 12 | 12 | 12 | 12 | 17 | 6 | 6 | 11 | 12 | - |  | 5 (1) |
| Lmi | 24 | 11 | 11 | 11 | 3 | 18 | 7 | 7 | 12 | 13 | 11 | - | 2 (2) |
| Total |  |  |  |  |  |  |  |  |  |  |  |  | 99 (19) |

Abbreviations are: LruNT = *L. ruficaudatus* (north of Tsiribihina); LruST = *L. ruficaudatus* (south of Tsiribihina); LruSB = *L. ruficaudatus* (south of Betsiboka); Led = *L. edwardsi*; Lmu = *L. mustelinus*; LdoAN = *L. dorsalis* (Ambanja/ Nosy Be); LdoS = *L. dorsalis* (Sahamalaza Peninsula); Lse = *L. septentrionalis*; Lan = *L. ankaranensis*; Lle = *L. leucopus*; Lmi = *L.* *microdon*; n = number of individuals karyotyped from 1975 till 2005, in brackets with R-banding.
