# Supplementary material for: Molecular phylogeny and taxonomic revision of the sportive lemurs (Lepilemur, Primates)
Source: BMC Evol Biol. 2006 Feb 23;6:17. doi: 10.1186/1471-2148-6-17 (PMC1397877; doi:10.1186/1471-2148-6-17)
Supplement: Additional File 3 — A table showing minimum and maximum uncorrected pairwise genetic differences (in %) within and among analysed species and populations based on complete mitochondrial cytochrome b sequence data. [file 1471-2148-6-17-S3.doc]

**Table 3:** Minimum and maximum uncorrected pairwise genetic differences (in %) within and among analysed species and populations based on complete mitochondrial cytochrome b sequence data

|  | **1** | **2** | **3** | **4** | **5** | **6** | **7** | **8** | **9** | **10** | **11** |
| --- | --- | --- | --- | --- | --- | --- | --- | --- | --- | --- | --- |
| **1 *L. leucopus*** | - |  |  |  |  |  |  |  |  |  |  |
| **2 *L. ruficaudatus***  (south of Tsiribihina) | 10.26-10.70 | 0.00-1.84 |  |  |  |  |  |  |  |  |  |
| **3 *L. ruficaudatus***  (north of Tsiribihina) | 10.44-10.70 | 6.15-7.11 | 0.00-2.28 |  |  |  |  |  |  |  |  |
| **4 *L. ruficaudatus***  (south of Betsiboka) | 11.23-11.32 | 7.11-7.63 | 5.88-6.75 | 0.00-0.26 |  |  |  |  |  |  |  |
| **5 *L. edwardsi*** | 12.54-12.63 | 12.46-12.98 | 11.75-12.02 | 12.46-12.63 | 0.00-0.09 |  |  |  |  |  |  |
| **6 *L. microdon*** | 11.40 | 11.75-12.46 | 11.32-11.84 | 12.28-12.72 | 9.21-9.47 | 0.00-0.53 |  |  |  |  |  |
| **7 *L. dorsalis***  (Sahamalaza Peninsula) | 13.07-13.42 | 11.23-12.02 | 11.58-12.28 | 12.46-13.07 | 12.02-12.54 | 10.97-11.40 | 0.00-1.05 |  |  |  |  |
| **8 *L. dorsalis***  (Ambanja/Nosy Be) | 12.63-12.72 | 10.97-11.93 | 11.49-11.67 | 10.97-11.32 | 10.70-10.79 | 10.26-10.53 | 5.18-5.88 | 0.00-0.26 |  |  |  |
| ***9 L. ankaranensis*** | 12.54-12.72 | 10.44-11.14 | 10.88-11.75 | 10.97-11.32 | 10.44-11.14 | 10.00-10.40 | 4.56-5.35 | 2.90-3.60 | 0.00-1.58 |  |  |
| **10 *L. septentrionalis*** | 13.16-13.86 | 11.14-12.72 | 10.79-12.02 | 12.46-12.63 | 10.70-11.58 | 9.83-10.79 | 9.30-11.14 | 7.46-8.95 | 7.37-9.39 | 0.00-1.58 |  |
| **11 *L. mustelinus*** | 15.44 | 14.47-15.35 | 14.82-15.61 | 15.61-15.97 | 16.23-16.58 | 16.14-16.40 | 16.40-16.82 | 16.05-16.23 | 15.35-15.70 | 15.88-16.49 | 0.61 |
