# Supplementary material for: Molecular phylogeny and taxonomic revision of the sportive lemurs (Lepilemur, Primates)
Source: BMC Evol Biol. 2006 Feb 23;6:17. doi: 10.1186/1471-2148-6-17 (PMC1397877; doi:10.1186/1471-2148-6-17)
Supplement: Additional File 4 — A table showing number of diagnostic characters as obtained from population aggregation analysis (PAA). [file 1471-2148-6-17-S4.doc]

**Table 4:** Number of diagnostic characters as obtained from population aggregation analysis (PAA)

|  | Lle | LruST | LruNT | LruSB | Led | Lmi | LdoS | LdoAN | Lan | Lse | Lmu |
| --- | --- | --- | --- | --- | --- | --- | --- | --- | --- | --- | --- |
| Lle | - |  |  |  |  |  |  |  |  |  |  |
| LruST | 115 | - |  |  |  |  |  |  |  |  |  |
| LruNT | 115 | 68 | - |  |  |  |  |  |  |  |  |
| LruSB | 124 | 78 | 64 | - |  |  |  |  |  |  |  |
| Led | 139 | 140 | 131 | 138 | - |  |  |  |  |  |  |
| Lmi | 125 | 131 | 125 | 135 | 101 | - |  |  |  |  |  |
| LdoS | 146 | 126 | 130 | 138 | 134 | 121 | - |  |  |  |  |
| LdoAN | 141 | 124 | 128 | 122 | 119 | 114 | 57 | - |  |  |  |
| Lan | 142 | 117 | 123 | 123 | 115 | 111 | 50 | 32 | - |  |  |
| Lse | 145 | 124 | 119 | 130 | 116 | 107 | 102 | 81 | 82 | - |  |
| Lmu | 171 | 163 | 165 | 174 | 183 | 179 | 183 | 179 | 172 | 173 | - |

Abbreviations are: Lle = *L. leucopus*; LruST = *L. ruficaudatus* (south of Tsiribihina); LruNT = *L. ruficaudatus* (north of Tsiribihina); LruSB = *L. ruficaudatus* (south of Betsiboka); Led = *L. edwardsi*; Lmi = *L.* *microdon*; LdoS = *L. dorsalis* (Sahamalaza Peninsula); LdoAN = *L. dorsalis* (Ambanja/Nosy Be); Lan = *L. ankaranensis*; Lse = *L. septentrionalis*; Lmu = *L. mustelinus*.
