# Supplementary material for: Molecular phylogeny and taxonomic revision of the sportive lemurs (Lepilemur, Primates)
Source: BMC Evol Biol. 2006 Feb 23;6:17. doi: 10.1186/1471-2148-6-17 (PMC1397877; doi:10.1186/1471-2148-6-17)
Supplement: Additional File 5 — A table showing morphometric measurements for the L. aeeclis syntype skull [file 1471-2148-6-17-S5.doc]

**Table 5:** Morphometric measurements for the *L. aeeclis* syntype skull

| **Skull (syntype UM 2003-Lem-100)** | | | **mm** |
| --- | --- | --- | --- |
| skull length | | | 59.0 |
| zygomatic width | | | 40.0 |
| bicanine width | | | 14.5 |
| facial length | | | 29.5 |
| maxillary toothrow | | | 21.8 |
| mandibular toothrow | | | 24.1 |
| orbital diameter min (average left and right) | | | 14.3 |
| orbital diameter max (average left and right) | | | 15.1 |
| postorbital width | | | 20.4 |
| **mandibel** | | | **mm** |
| hight of ramus mandibularis (average left and right) | | | 23.7 |
| length of mandible (average left and right) | | | 45.4 |
| **dentition** | | | **mm** |
| tooth comb length | | | 6.3 |
| tooth comb basal width | | | 4.7 |
| tooth apical width | | | 3.9 |
| premolar tooth row length | | | 10.8 |
| molar tooth row length | | | 13.0 |
| upper dentition LxW | mm | lower dentition LxW | mm |
| C | 4.7 x 2.2 |  |  |
| P2 | 3.7 x 2.2 | P2 | 4.5 x 2.2 |
| P3 | 3.5 x 3.0 | P3 | 3.8 x 2.2 |
| P4 | 3.2 x 3.4 | P4 | 3.7 x 2.2 |
| M1 | 4.3 x 4.3 | M1 | 4.3 x 2.9 |
| M2 | 4.4 x 4.5 | M2 | 4.2 x 3.0 |
| M3 | 3.7 x 3.9 | M3 | 4.6 x 2.7 |
