# Supplementary material for: Molecular phylogeny and taxonomic revision of the sportive lemurs (Lepilemur, Primates)
Source: BMC Evol Biol. 2006 Feb 23;6:17. doi: 10.1186/1471-2148-6-17 (PMC1397877; doi:10.1186/1471-2148-6-17)
Supplement: Additional File 6 — A table showing morphometric measurements of L. randrianasoli and L. aeeclis in comparison with L. ruficaudatus. [file 1471-2148-6-17-S6.doc]

**Table 6:** Morphometric measurements of *L. randrianasoli* and *L. aeeclis* in comparison with *L. ruficaudatus*

|  | ***L. randrianasoli* (n=9)**  **Locality: Andramasay** | | ***L. ruficaudatus* (n=45)**  **Locality: Kirindy/CFPF** | | ***L. aeeclis* (n=5)**  **Locality: Antafia/Anjahamena** | |
| --- | --- | --- | --- | --- | --- | --- |
|  | **female (n=4)** | **male (n=5)** | **female (n= 24)** | **male (n=21)** | **female (n=2)** | **male (n=3)** |
| ^1^ body mass (g)** | 793 (740 - 880) | 717 (660 - 760) | 803 (670 - 930) | 806 (712 - 896) | 909 (845 - 972) | 868 (763 - 940) |
| ^2^ head-body length (mm) |  |  | 309 (289 - 335) | 308 (287 - 344) | 300 (295 - 305) | 303 (285 - 315) |
| ^3^ head length (mm) | 63.6 (60.8 - 66.2) | 63.9 (63.1 - 66.0) | 62.2 (57.8 - 65.3) | 62.8 (60.4 - 65.8) | 63.0 (62.0 - 64.0) | 63.0 (60.0 - 65.0) |
| ^4^ head width (mm)** | 37.4 (36.5 - 38.8) | 38.0 (37.3 - 38.8) | 39.2 (37.2 - 42.0) | 39.7 (37.3 - 43.0) | 39.0  (n=1) | 43.3 (37.0 - 48.0) |
| ^5^ lower hind leg length (mm) | 102 (100 - 105) | 101 (97 - 107) |  |  | 101 (100 - 102) | 99 (94 - 105) |
| ^6^ hind foot length (mm)*** | 78.8 (78 - 80) | 78.2 (74 - 81) | 68.7 (63 - 74) | 68.2 (63 - 74) | 79.0 (79 - 79) | 77.7 (75 .- 80) |
| ^7^ tail length (mm) | 240 (220 - 253) | 226 (209 - 244)  (n=4) | 249 (203 - 281) | 238 (209 - 270)  (n=20) | 255 (250 - 260) | 252 (240 - 260) |

^1^ANOVA body mass: site F_2,53_ = 7.18, p<0.01; sex:F_1,53_ = 2.45, ns; site x sex F_2,53_ = 1.63, ns

^2^ANOVA head-body length: site F_1,46_ = 1.19, ns; sex:F_1,46_ = 0.03, ns; site x sex F_1,46_ = 0.11, ns

^3^ANOVA head length: site F_2,53_ = 1.78, ns; sex:F_1,53_ = 0.21, ns; site x sex F_2,53_ = 0.08, ns

^4^ANOVA head width: site F_2,52_ = 6.19, p<0.01; sex:F_1,52_ = 6.06, p<0.05; site x sex F_2,52_ = 1.83, ns

^5^ANOVA lower hind leg length: site F_1,10_ = 0.69, ns; sex:F_1,10_ = 0.82, ns; site x sex F_1,10_ = 0.03, ns

^6^ANOVA hind foot length: site F_2,53_ = 59.53, p<0.001; sex:F_1,53_ = 0.49, ns; site x sex F_2,53_ = 0.04, ns

^7^ANOVA tail length: site F_2,51_ = 2.27, ns; sex:F_1,51_ = 1.91, ns; site x sex F_2,51_ = 0.15, ns
