# Supplementary material for: Molecular phylogeny and taxonomic revision of the sportive lemurs (Lepilemur, Primates)
Source: BMC Evol Biol. 2006 Feb 23;6:17. doi: 10.1186/1471-2148-6-17 (PMC1397877; doi:10.1186/1471-2148-6-17)
Supplement: Additional File 7 — A table showing morphometric measurements for L. sahamalazensis in comparison to L. dorsalis. [file 1471-2148-6-17-S7.doc]

**Table 7:** Morphometric measurements for *L. sahamalazensis* in comparison to *L. dorsalis*

|  | ***L. sahamalazensis* (n=6)**  **Locality: Ankarafa forest** | | ***L. dorsalis* (n=18)**  Locality: Nosy Be | |
| --- | --- | --- | --- | --- |
|  | **males (n=2)** | **females (n=4)** | **males (n=9)** | **females (n=9)** |
| ^1^body mass (g)* | 691 (687-694) | 787 (740 – 892) | 817 (700 – 900) | 923 (660 – 1110) |
| ^2^head-body length (mm)*** | 258 (252 – 264) | 259 (254 – 266) | 305 (250 – 340) | 319 (280 – 355) |
| head length (mm) | 56.5 (56.2 – 56.8) | 57.1 (55.6 – 58.4) |  |  |
| head width (mm) | 35.2 (34.6 – 35.8) | 35.1 (34.1 – 36.8) |  |  |
| ^3^tibia length (mm)*** | 103.0 (102 – 104) | 100.5 (98 – 105) | 86.4 (80 – 93) | 87.8 (81 – 102) |
| ^4^tarsus length (mm) | 48.2 (48 – 49) | 49.3 (47 – 52) | 48.2 (45 – 52) | 47.4 (45 – 51) |
| ^5^tail length (mm) | 260  (n=1) | 271 (267 – 274)  (n=2) | 257 (244 – 270)  (n=8) | 249 (230 – 270)  (n=8) |
| ^6^ear length (mm) | 25.4 (24 – 27) | 25.3 (23 – 28) | 22.6 (21 – 25) | 23.6 (19 – 31)  (n=8) |

^1^ANOVA body mass: site F_1,20_ = 5.91, p<0.05; sex:F_1,20_ = 3.51, p<0.08; site x sex F_1,20_ = 0.01, ns

^2^ANOVA head-body length: site F_1,20_ = 17.31, P<0.001; sex:F_1,20_ = 0.37, ns; site x sex F_1,20_ = 0.30, ns

^3^ANOVA tibia length: site F_1,20_ = 29.16, p<0.001; sex:F_1,20_ = 0.04, ns; site x sex F_1,20_ = 0.51, ns

^4^ANOVA tarsus length: site F_1,20_ = 0.70, ns; sex:F_1,20_ = 0.02, ns; site x sex F_1,20_ = 0.73, ns

^5^ANOVA tail length: site F_1,15_ = 2.13, ns; sex:F_1,15_ = 0.02, ns; site x sex F_1,15_ = 1.29, ns

^6^ANOVA tail length: site F_1,19_ = 3.10, p<0.10; sex:F_1,19_ = 0.11, ns; site x sex F_1,19_ = 0.17, ns
