# Supplementary material for: Molecular phylogeny and taxonomic revision of the sportive lemurs (Lepilemur, Primates)
Source: BMC Evol Biol. 2006 Feb 23;6:17. doi: 10.1186/1471-2148-6-17 (PMC1397877; doi:10.1186/1471-2148-6-17)
Supplement: Additional File 8 — A table showing classification of sportive lemurs. [file 1471-2148-6-17-S8.doc]

Table 8: Classification of sportive lemurs

| Family LEPILEMURIDAE | |
| --- | --- |
| Genus *Lepilemur* I. Geoffroy, 1851 | |
| Species | Distribution |
| *L. aeeclis** sp. nov. | West Madagascar, south of Betsiboka River, southern range limit not yet determined |
| L. ankaranensis  Rumpler and Albignac, 1975 | North Madagascar, Ankarana Massif |
| *L. dorsalis*  Gray, 1870 | North-West Madagascar |
| *L. edwardsi*  Forsyth Major, 1894 | North-West Madagascar, Mahajanga region |
| *L. leucopus*  Forsyth Major, 1894 | South Madagascar, south of Onilahy River |
| *L. microdon*  Forsyth Major, 1894 | East Madagascar, south of Tamatave |
| *L. mustelinus*  I. Geoffroy, 1851 | East Madagascar, north of Andevoranto |
| *L. randrianasoli** sp. nov. | West Madagascar, north of Tsiribihina River, northern range limit not yet determined |
| *L. ruficaudatus*  A. Grandidier, 1867 | West Madagascar, south of Tsiribihina River, possibly south to Onilahy River |
| *L. sahamalazensis** sp. nov. | North-West Madagascar, Sahamalaza Peninsula, exact range limits not yet determined |
| *L. septentrionalis*  Rumpler and Albignac, 1975 | Far North of Madagascar |
